# Nano‐Topography Enhanced Topological‐Cell‐Analysis in Radiation‐Therapy

**DOI:** 10.1002/adhm.202405187

**Published:** 2025-03-22

**Authors:** Francesca Pagliari, Maria‐Francesca Spadea, Pierre Montay‐Gruel, Anggraeini Puspitasari‐Kokko, Joao Seco, Luca Tirinato, Angelo Accardo, Francesco De Angelis, Francesco Gentile

**Affiliations:** ^1^ Division of BioMedical Physics in Radiation Oncology German Cancer Research Center 69120 Heidelberg Germany; ^2^ Institute of Biomedical Engineering Karlsruhe Institute of Technology (KIT) 76131 Karlsruhe Germany; ^3^ Radiation Oncology Department Iridium Netwerk Antwerp 2610 Belgium; ^4^ Antwerp Research in Radiation Oncology (AreRO) Center for Oncological Research (CORE) University of Antwerp Antwerp 2020 Belgium; ^5^ Research and Development ‐ HollandPTC Delft University of Technology Delft The Netherlands; ^6^ Department of Physics and Astronomy Heidelberg University Im Neuenheimer Feld 227 69120 Heidelberg Germany; ^7^ Department of Medical and Surgical Sciences University Magna Graecia of Catanzaro Catanzaro 88100 Italy; ^8^ Department of Precision and Microsystems Engineering Faculty of Mechanical Engineering Delft University of Technology Mekelweg 2 Delft 2628 CD The Netherlands; ^9^ Plasmon nano‐technologies Italian Institute of Technology Genova 16163 Italy; ^10^ Nanotechnology Research Center Department of Experimental and Clinical Medicine University of Magna Graecia of Catanzaro Catanzaro 88100 Italy

**Keywords:** AI, biomaterials, nano‐topography, networks science, radiation‐therapy, Raman phenotyping, scaffolds, topology

## Abstract

Radiotherapy (RT) is a cancer treatment technique that involves exposing cells to ionizing radiation, including X‐rays, electrons, or protons. RT offers promise to treat cancer, however, some inherent limitations can hamper its performance. Radio‐resistance, whether innate or acquired, refers to the ability of tumor cells to withstand treatment, making it a key factor in RT failure. This perspective hypothesizes that nanoscale surface topography can impact on the topology of cancer cells network under radiation, and that this understanding can possibly advance the assessment of cell radio‐resistance in RT applications. An experimental plan is proposed to test this hypothesis, using cancer cells exposed to various RT forms. By examining the influence of 2D surface and 3D scaffold nanoscale architecture on cancer cells, this approach diverges from traditional methodologies, such as clonogenic assays, offering a novel viewpoint that integrates fields such as tissue engineering, artificial intelligence, and nanotechnology. The hypotheses at the base of this perspective not only may advance cancer treatment but also offers insights into the broader field of structural biology. Nanotechnology and label‐free Raman phenotyping of biological samples are lenses through which scientists can possibly better elucidate the structure‐function relationship in biological systems.

## Graphical abstract

Radiotherapy, a treatment that implies exposition of cells to radiation, offers promise to treat cancer. Here, it is hypothesized that the nanoscale details of a scaffold can amplify the response of cells to radiation, measured through topological variables, providing ways to assess the efficacy of Radiotherapy treatment.

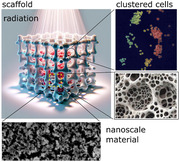

## 1 Introduction

**Figure 1:**
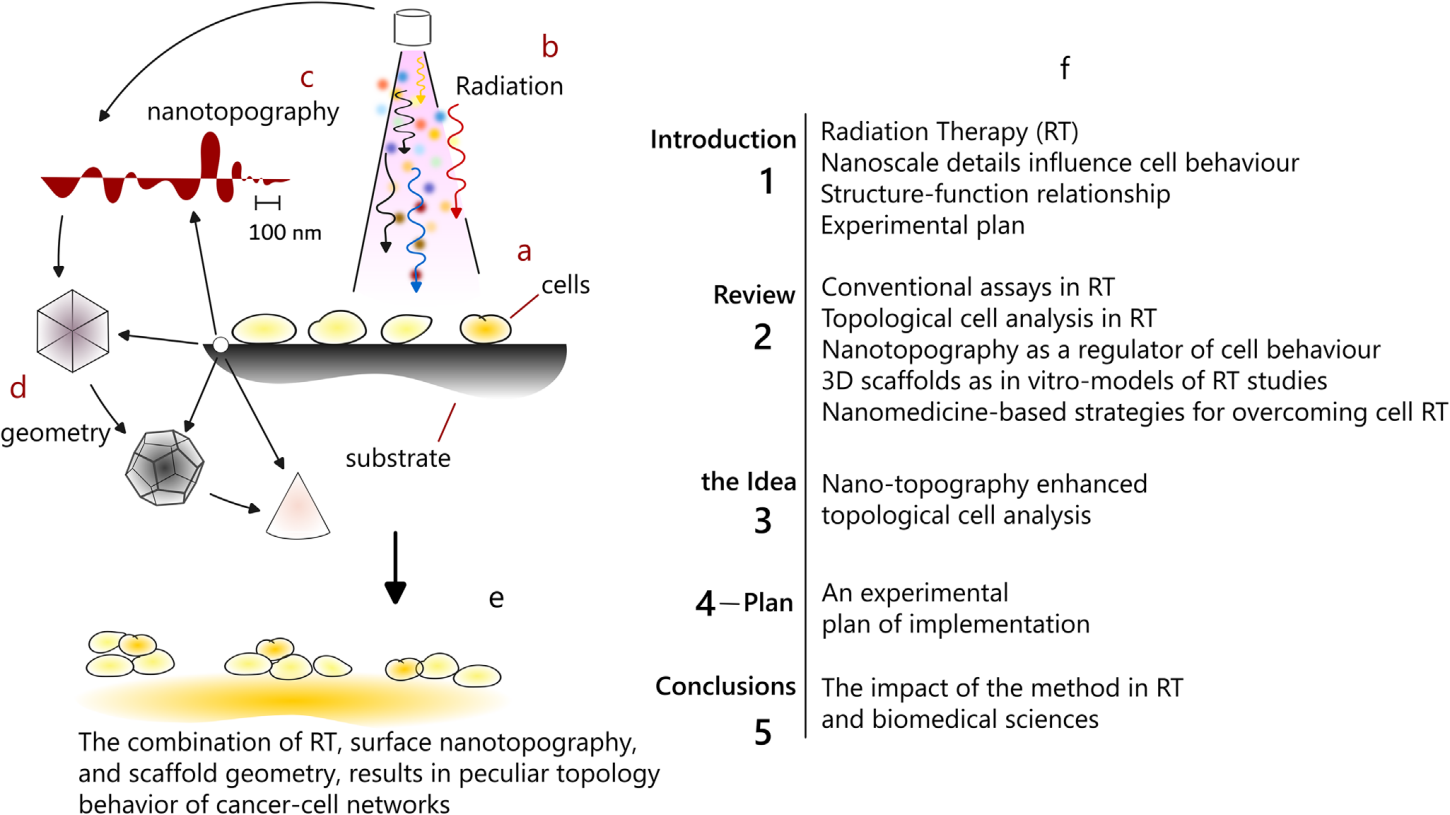
*Schematic representation of the proposed approach detailed in the perspective*. Cancer cells are cultured in biomimetic scaffolds a) and exposed to radiation b). The scaffold's surface nano‐topography c) and overall architecture d) can be precisely engineered using nanotechnology. The interplay between surface nano‐topography, scaffold geometry, and radiation exposure leads to the organization of cells into specific architectures e). A topological analysis of these cellular networks provides insights into their biological behavior and potential resistance to radiotherapy. The inset f) summarizes the structure of this work, which includes i) an introduction, ii) a review section, iii) a discussion of the proposed approach—nano‐topography‐enhanced topological cell analysis, iv) an experimental implementation plan, and v) a conclusion.

This perspective proposes to explore the combined effects of surface topography, scaffold geometry, and RT treatment on the topology of cancer cell networks, examining how these responses are modulated by the intrinsic radiation resistance (RR) of cells (**Figure** **1**). The work is structured as follows.

In the *introduction*, we first briefly describe how RT works, and suggest that surface‐enhanced topological cell analysis can be a powerful tool for RT assessment (1.1). Then, we discuss the notion that nano‐topography can efficiently guide cell behavior, and the spatial arrangement of cells, i.e., structure (1.2). Subsequently, we recall that, in biological systems, structure and function are correlated (1.3) providing examples of how, by measuring the internal architecture of a system one can have an indication of how that system works (1.4). In the last section of the introduction, we outline a possible experimental plan necessary to develop these ideas (1.5).

Thereafter, we *review* the existing body of literature related to RT, either pertaining to conventional methods of analysis, such clonogenic assays (2.1), or to a recently introduced technique that leverages cell‐network topology (2.2). Then, we critically review reports on nano‐topography as a regulator of cell behavior (2.3) and comment on the use of 3D scaffolds as an in‐vitro‐model for RT studies (2.4). The final section of the review focuses on analyzing current nanomedicine strategies to overcome cell RR (2.5).

In the next section, we introduce the object of this perspective, i.e., *nano‐topography enhanced topological cell analysis*, and substantiate the claim that this new field of study can improve over the existing methods of RT assessment, in terms of precision, sensitivity, accuracy, and cost‐effectiveness (3). In doing so, we describe possible outcomes related to the research, including technology, scientific and social outcomes. A possible *experimental plan of implementation* of this idea is briefly outlined in a separate study design section (4). In the *conclusions*, we discuss the potential impact of the concepts outlined in this perspective on cancer care and the broader field of biomedical sciences (5).

### 1.1 Radiation Therapy ‐ RT

RT is a cancer treatment approach that involves exposing cancerous tissues and organs to ionizing radiation, such as X‐rays, electrons, or protons. This radiation damages the genetic material and molecular structure of cells, disrupting their functions, including proliferation and growth, altering their cellular interactions, and ultimately leading to their death.^[^ ^1^, ^2^ ^]^ In modern clinical practice, around half of all cancer patients undergo RT, either as a standalone treatment or in combination with chemotherapy and surgery.^[^ ^3^ ^]^ Although radiotherapy holds great potential for cancer treatment, certain inherent limitations can reduce its effectiveness. RR, whether innate or acquired, enables tumor cells to withstand treatment through various mechanisms. These include enhanced DNA damage repair, autophagy, adaptation to hypoxia, and the presence of cancer stem cells, all of which are linked to alterations in the tumor microenvironment.^[^ ^4^, ^5^ ^]^ RR is a key contributor to the failure of RT. Developing and strengthening techniques and methods to evaluate cell sensitivity to internal or external radiation fields is thus critically important. *The aim of this perspective is to propose a method for assessing the RR of cancer cells and to outline a study design for its implementation*. In particular, the research is designed to explore the influence of the nanoscale architecture of 2D surfaces and 3D scaffolds on the *topology* of cancer cell networks under radiation. The idea behind this perspective stems from the observation that external radiation fields alter cellular DNA, influencing the collective behavior of cellular systems. Here, we hypothesize that nano‐topography can amplify these effects. This approach leverages detailed knowledge of cell anatomy and spatial organization within colonies to assess biological characteristics, using topological measures instead of traditional clonogenic assays for evaluating RT treatment effectiveness.

### 1.2 Nano‐Topography Drives Cell Assembly

The formation of multicellular structures is governed by adhesion forces, which regulate interactions both between individual cells and between cells and their surrounding environment. This interaction determines the evolution of cell systems and guides them toward ordered structures.^[^ ^6^, ^7^, ^8^ ^]^ Nano‐topography, in particular, has been identified as a critical factor in directing cell fate at the bio‐interface,^[^ ^9^, ^10^, ^11^, ^12^ ^]^ making it a significant area of interest in tissue engineering, biosensors, the analysis of neurodegenerative disorders. Studies^[^ ^13^, ^14^, ^15^ ^]^ have demonstrated that cells cultured on surfaces with nanoscale motifs tend to arrange into clusters with high clustering and short average path lengths. *Thanks to this peculiar architecture, similar structures exhibit enhanced computational capabilities compared to cells randomly distributed on a surface without any special layout*. Thus, in the context of this perspective, nano‐topography plays a major role because it represents a factor – a parameter – through which one can possibly control the shape of biological aggregates and, notably, their function.

The organization of cells into networks with specific topologies enhances overall network performance—much like how an organism's functionality depends more on the collective cooperation of its elements than on their individual properties. In the brain, complex functions such as memory, language, and thought emerge from the continuous exchange of signals (action potentials). The dynamic positioning of signal sources and drains influences the distances and paths these signals must travel, directly impacting their efficiency. Similar to the traveling salesman problem, system performance is highly sensitive to component layout, a principle studied in topology and network science. This fundamental relationship explains why computational power is tied to network topology. While the correlation between structure and function in biological systems has long been recognized, a seminal study in the early 1980s^[^ ^16^ ^]^ first demonstrated how neural network computation depends on collective behavior—a discovery that earned a Nobel Prize in physics 45 years later. More recently, researchers have investigated how the cell‐material interface regulates information flow in biological networks, using both experimental^[^ ^14^, ^15^, ^17^ ^]^ and numerical^[^ ^18^ ^]^ approaches.

### 1.3 In Biological Systems, Structure and Function are Correlated

**Figure 2:**
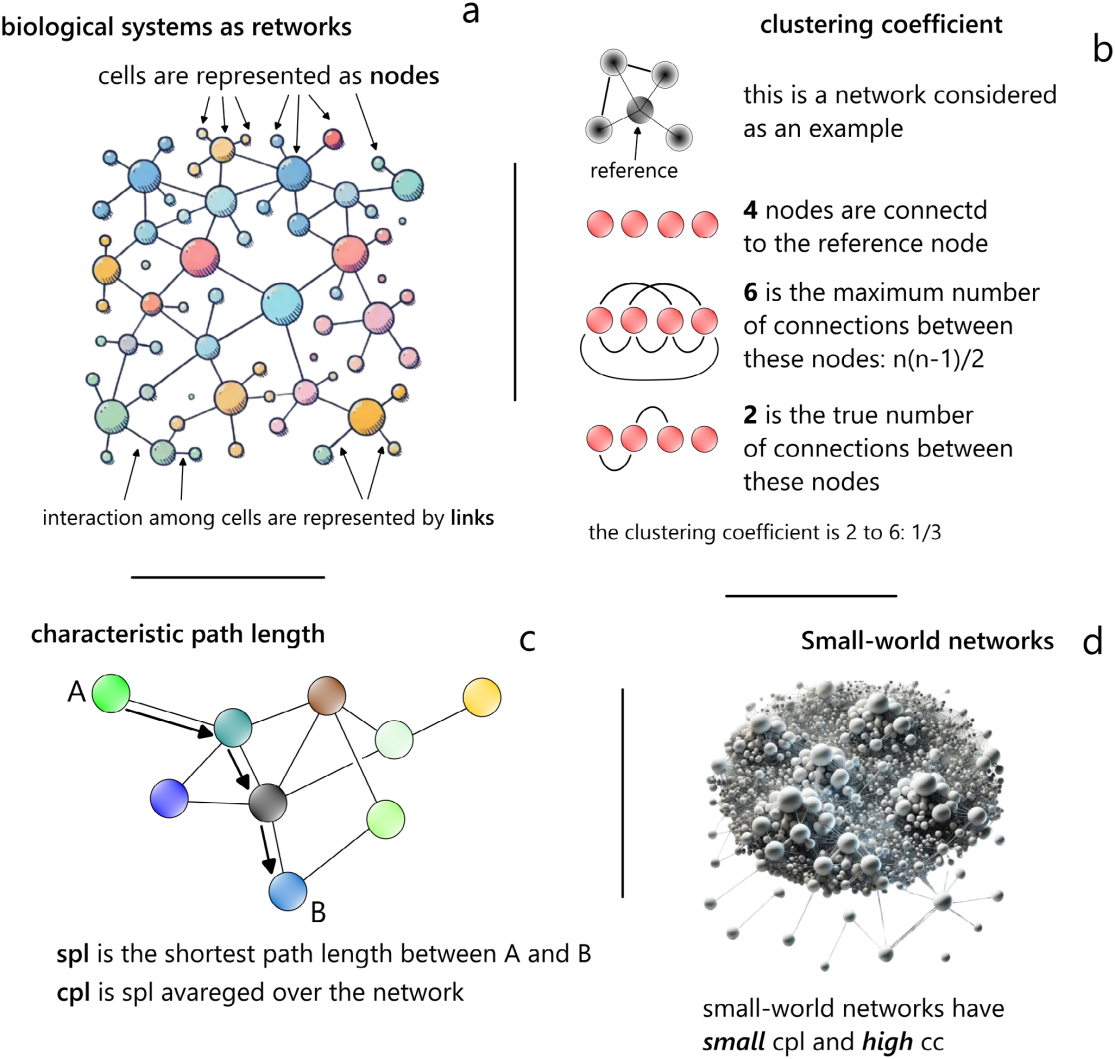
*A graphical representation of networks science*. The study of networks, known as network science, has gained prominence across various disciplines as a tool for analyzing complex systems: including social, biological, and technological systems.^[^ ^26^, ^28^, ^30^, ^31^, ^32^ ^]^ In particular, biological systems can be efficiently described as networks a). We can use different parameters to describe networks, including the network degree, the clustering coefficient b), the characteristic path length c), and the small world coefficient d). The network degree (k) represents the average number of links per node in a graph. The clustering coefficient (*cc*) measures graph connectivity, ranging from 0 to 1. It quantifies the proportion of existing links among the nodes surrounding a given element compared to the total possible connections among them, averaged across the entire network. The characteristic path length (*cpl*) refers to the average shortest path between network elements. Among these parameters, the small‐world coefficient (*SW*) is particularly significant. It is derived from the combination of cc and *cpl*, representing the balance between network connectivity (*cc*) and proximity (*cpl*) relative to a random graph of the same size.^[^ ^33^ ^]^ – SW is non‐dimensional. Small‐world networks with SW > 1 are topologically biased to enhance local connectivity,^[^ ^28^, ^31^, ^32^ ^]^ which in turn optimizes system's performance.^[^ ^14^, ^18^, ^34^, ^35^, ^36^, ^37^ ^]^ The study of small‐world networks has recently gained momentum because many real systems, phenomena and processes can be modelled after this network topology.

The way in which cells of a system are arranged in the space, and the way in which they are interconnected, govern the functionality and overall behavior of biological systems.^[^ ^16^, ^19^, ^20^ ^]^ The peculiar characteristics of similar systems rest more on the cooperation of numerous cells at different organizational levels, and less on the attributes of individual cells.^[^ ^21^, ^22^, ^23^ ^]^ (This is, notably, the definition of complex systems.^[^ ^24^, ^25^, ^26^, ^27^ ^]^) Thus, studying and understanding the functions of biological systems inevitably requires a deep comprehension of their structure. This includes examining their architecture, form, the connections between individual components, and how these components communicate. To efficiently do this, it is convenient representing systems as networks.^[^ ^28^, ^29^ ^]^ In simple terms, a network (or a graph) is a set of objects that are connected together. The objects of a network, in turn, are regarded as nodes: the connections between the nodes are called edges or links. Networks science is the discipline that studies networks. In the context of networks science, notably, correlation implies causation: objects of a network that are connected do also interact (**Figure** **2**). This also suggests that biological systems can be modeled as networks, where the nodes represent system elements and the links describe the interactions between them. One key advantage of networks is that they can be quantified. Networks’ *topology* defines the way a network is shaped and how its internal components are associated. There exist quantitative metrics that precisely measure topology, and make networks a universal tool confidently used in different areas across biology, ecology, and social science.^[^ ^28^, ^29^ ^]^

### 1.4 Examples of Structure‐Function Relationships in Biological Systems and Vision

One notable example of a biological system where structure and function are manifestly correlated is the brain: a tightly interweaving network of neural cells. In this system, the topological structure of the network is responsible for increased computational ability, low energy consumption, and non‐deterministic functions like language and memory. The complexity of human behavior is driven less by the specialization of individual nerve cells and more by the formation of intricate networks composed of a large number of these cells.^[^ ^18^, ^26^, ^38^, ^39^ ^]^ Theories like the integrated information theory propose that consciousness itself emerges from the way information is processed within a system, indicating a direct correlation between interconnectedness and higher cognitive functions.^[^ ^40^ ^]^ Understanding how individual cells interact to form complex systems is paramount for fields like tissue engineering and regenerative medicine, as it offers insights into the formation of in‐vitro models, the analysis of neuro‐degenerative diseases, the design of efficient prosthesis implants, and neuromorphic engineering. The exploration of biological systems through the lens of their internal architecture, topology, and spatial layout reveals a fascinating interplay between structure and function. The influence of 3D scaffold architecture on cell network topology opens up new avenues in understanding how biological systems self‐organize and function. This insight not only enhances our comprehension of natural biological processes but also guides the development of synthetic systems and therapeutic interventions. The implications of these findings are vast, extending from tissue engineering to the intricate workings of the human brain, showing the profound impact of architecture and topology on the functionality of biological systems.

It is important to note that both the geometry of 2D surfaces and the architecture of 3D scaffolds play a crucial role in shaping cell network topology. A number of examples, conveniently reported in a separate Supporting Information 1, demonstrate the effectiveness of bidimensional surfaces in regulating of cell‐assembly. Nanoscale 2D surfaces serve as the crucial link between traditional 2D cell culture models and more advanced 3D templates. They provide the necessary complexity for cell colony growth while enhancing physiological relevance, drug response accuracy, and predictive power—hallmarks of 3D cell models.^[^ ^41^ ^]^

### 1.5 Outline of the Work

**Figure 3:**
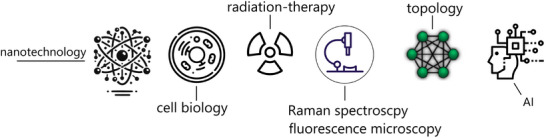
*Interdisciplinary approach*. Various disciplines have to be integrated to enhance our understanding of cancer RT.

This article postulates that cancer‐cell response to radiation can be modulated by an engineered micro‐environment. Moreover, we suggest that the topology of a biological system subjected to external radiation fields can be measured using the methods of networks science, and that such a topological measure can be indicative of the system's state. Throughout the text, stemming from a critical review of the existing body of literature on cancer cell RT, nano‐topography, and the development of 3D scaffolds for in vitro‐studies of biological problems, we provide evidence of this hypothesis. In addition, we sketch a possible experimental plan that can be used to test the hypothesis of nano‐topography enhanced topological‐cell‐analysis in RT. Such a plan involves exposing cancer cells to various RT modalities, in both conventional and ultra‐high dose rate settings. The architecture of the resulting cell networks, conveniently analyzed using Network Science and Artificial Intelligence, can possibly enable the construction of predictive models to forecast patient responses to radiation based on ex vivo radio‐sensitivity (RS) tests. Additionally, Raman spectroscopy and electrophysiological monitoring can be designed to examine the correlation between the topology of cell networks and the phenotypic traits expressed by the cells. By investigating the correlation between the topology of cell networks and the phenotypic traits expressed by the cells, this research aims to establish a link between structure and function of a biological system. This study‐design represents a pioneering approach, integrating nanotechnology, cell biology, radiation therapy, AI, and network science, to advance understanding of cancer treatment and response mechanisms (**Figure** **3**).

## 2 Literature Review

This section presents a thorough review of the existing body of literature related to i) RT assessment, ii) topological cell analysis, iii) nano‐topography driven cell assembly, iv) 3D scaffolds for in vitro‐models study of RT, and v) nanomedicine strategies in RT.

### 2.1 Consolidated Methods of Analysis of RT

State‐of‐the‐art techniques for assessing cell RR and RS include cell proliferation assays,^[^ ^42^ ^]^ the neutral comet assay,^[^ ^43^ ^]^ gamma‐H2AX immunofluorescence staining,^[^ ^44^ ^]^ and the clonogenic (colony formation) assay.^[^ ^45^ ^]^ Among these, the clonogenic assay is widely utilized in radiation biology, as it establishes a correlation between cell RS, survival, and clinical responses to RT in both tumor cell lines and patient‐derived tumor cells.^[^ ^46^ ^]^ This in‐vitro cell survival assay evaluates the capacity of a single cell to develop into a colony. In this method, cancer cells are seeded onto a substrate, supplemented with a cell culture medium, exposed to ionizing radiation, and observed over time. The plating efficiency and surviving fraction values obtained from this assay provide key insights into a cancer cell's ability to withstand radiation. Recognized as the gold standard for studying cancer cell responses to radiation, the clonogenic assay, however, is influenced by several factors, including cell type, doubling time, cell density, cellular interactions, and nutrient availability. In radiobiology studies using the clonogenic assay, each colony is traditionally treated as a *black box*, meaning the individual characteristics of cancer cells within each surviving colony are often overlooked. Colonies are typically considered as single entities, without accounting for intrinsic cellular variability. While conventional clonogenic assays primarily measure the number of surviving colonies post‐treatment, the technique has advanced over time, incorporating greater complexity. Recently, several research groups^[^ ^47^, ^48^ ^]^ have proposed using the morphological characteristics of cells as an additional parameter to assess their RR or RS.

### 2.2 Topological Cell Analysis of Radiation‐Therapy: A Paradigm Shift

In a recent study,^[^ ^49^ ^]^ researchers have investigated the relationship between the topological properties of lung cancer networks in 2D cultures and their response to ionizing radiation. In experiments in which the dose was varied up to 8 *Gy*, the study showed that the topological measures of cancer lung cell‐graphs also varied significantly. In the continuation of the research,^[^ ^50^ ^]^ the same team demonstrated that radiation in the 0 − 6 *Gy* range influences the topology of cancer cell lines different from cancer, including epithelial neuroglioma cells, PC3 bone metastasis of prostate cancer and urinary bladder cancer cells (a more detailed analysis of the correlation between external radiation dose and cancer cell network topology for different cell lines is provided in Supporting Information 2). Current clonogenic protocols^[^ ^45^ ^]^ aim to assess cell RS and RR by analyzing the number of cell colonies that form on a 2D substrate after irradiation.

However, the number of colonies in a sample is an estimate of how cells behave in response to radiotherapy. Methods devised in references^[^ ^49^, ^50^ ^]^ are an attempt to surpass the limitations found in conventional clonogenic assays.

**Figure 4:**
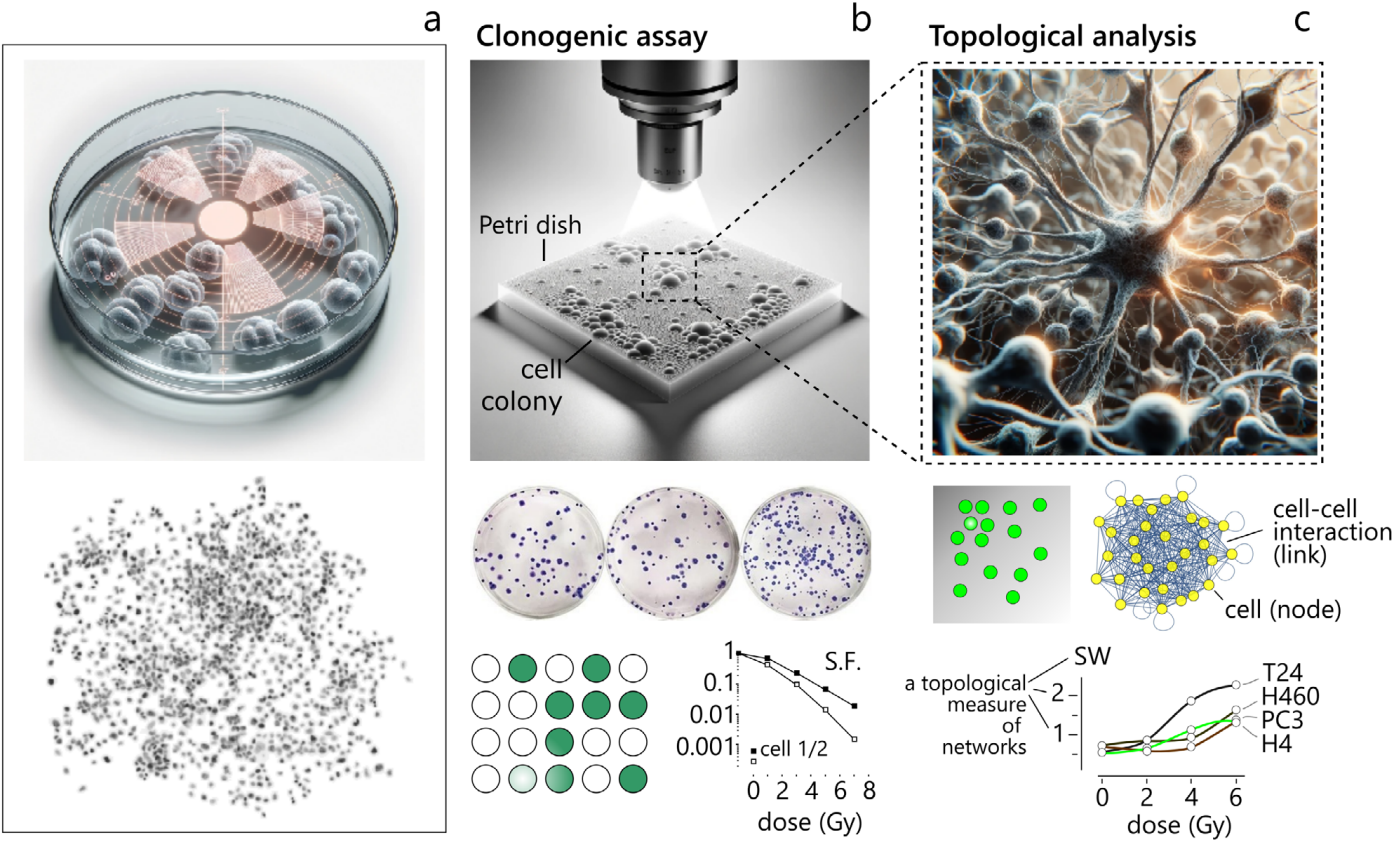
*Comparative assessment of RR techniques: clonogenic assay* versus *topological cell analysis*. Schematic representation of cells exposed to RT and their post‐exposure spatial organization, highlighting the importance of networks science for describing the impact of radiation on cell clustering and network formation a). Traditional clonogenic assays assess RR by counting the number of surviving colonies, without considering their internal spatial structure b). Topological cell analysis quantitatively characterizes cell‐network architecture using metrics such as clustering coefficient, characteristic path length, and small‐world coefficient. These network parameters provide a higher‐resolution measure of cellular response to RT by capturing the connectivity and organization of surviving cell populations. By integrating nano‐engineered surfaces, topological analysis can enhance sensitivity in detecting subtle alterations in cell behavior post‐irradiation, offering a novel approach for evaluating RR and tailoring treatment strategies c).

In these cited works, *topology* is utilized to explore the behavior of cancer cells exposed to radiation. The topological structure of a large number of cells within a colony is determined by the spatial arrangement of their nuclei, reflecting how cells are interconnected and organized. Compared to simple cell counting, topology provides a more comprehensive, detailed, and quantitative representation of the system (**Figure** **4**). Cancer cell graphs emerge from the interactions among all elements within a colony, encapsulating the maximum amount of information about the cellular system. As a result, network variables offer a robust and reliable means of describing how external factors, such as radiation, influence the system.

This concept can be promptly applied to clinical practice. Tumor tissue obtained through solid biopsy can be dissociated, and the resulting cancer cells cultured on a support. As these cell colonies develop, they can be exposed to progressively higher radiation doses and analyzed using the methods outlined in this study. The small‐world coefficient values derived from these samples will then be compared to those of non‐cancerous cells from the same tissue or organ. This comparison will help determine whether the patient's cells are radio‐sensitive or radio‐resistant and to what extent—critical factors in personalizing the most effective treatment strategy for the patient.

Thus, *topological cell analysis* offers promises to overcome the limitations of clonogenic assays. To place this work in context and substantiate the claims of the perspective, in a separate Supporting Information 3 it is thoroughly described how clonogenic assays work, especially pertaining to tissue culture plastic models –, i.e., still the gold standard for determining the RS of cells. This section examines the critical aspects of clonogenic assays, including their robustness and inter‐assay precision. Additionally, it presents a quantitative comparison of the sensitivity of clonogenic assays versus topological cell analysis, based on reported cases in the literature. The findings suggest that topological cell analysis, along with its future advancements, offers significant improvements over conventional clonogenic assays.

The image of cells in inset (a) is adapted under the terms of the CC‐BY Creative Commons Attribution 4.0 International license (https://creativecommons.org/licenses/by/4.0):^[^ ^49^ ^]^ Copyright 2022, published by Nature Portfolio. The clonogenic assay image in insets (b) is adapted under the terms of the CC‐BY Creative Commons Attribution 4.0 International license (https://creativecommons.org/licenses/by/4.0):^[^ ^49^ ^]^ Copyright 2020, published by Nature Portfolio. The measured values of small‐world coefficient as a function of external dose for the T24, H460, PC3 and H4 cell lines shown in inset (c), are adapted under the terms of the CC‐BY Creative Commons Attribution 4.0 International license (https://creativecommons.org/licenses/by/4.0):^[^ ^50^ ^]^ Copyright 2023, published by Springer.

### 2.3 Nano‐Topographical Control of Cell Assembly

**Figure 5:**
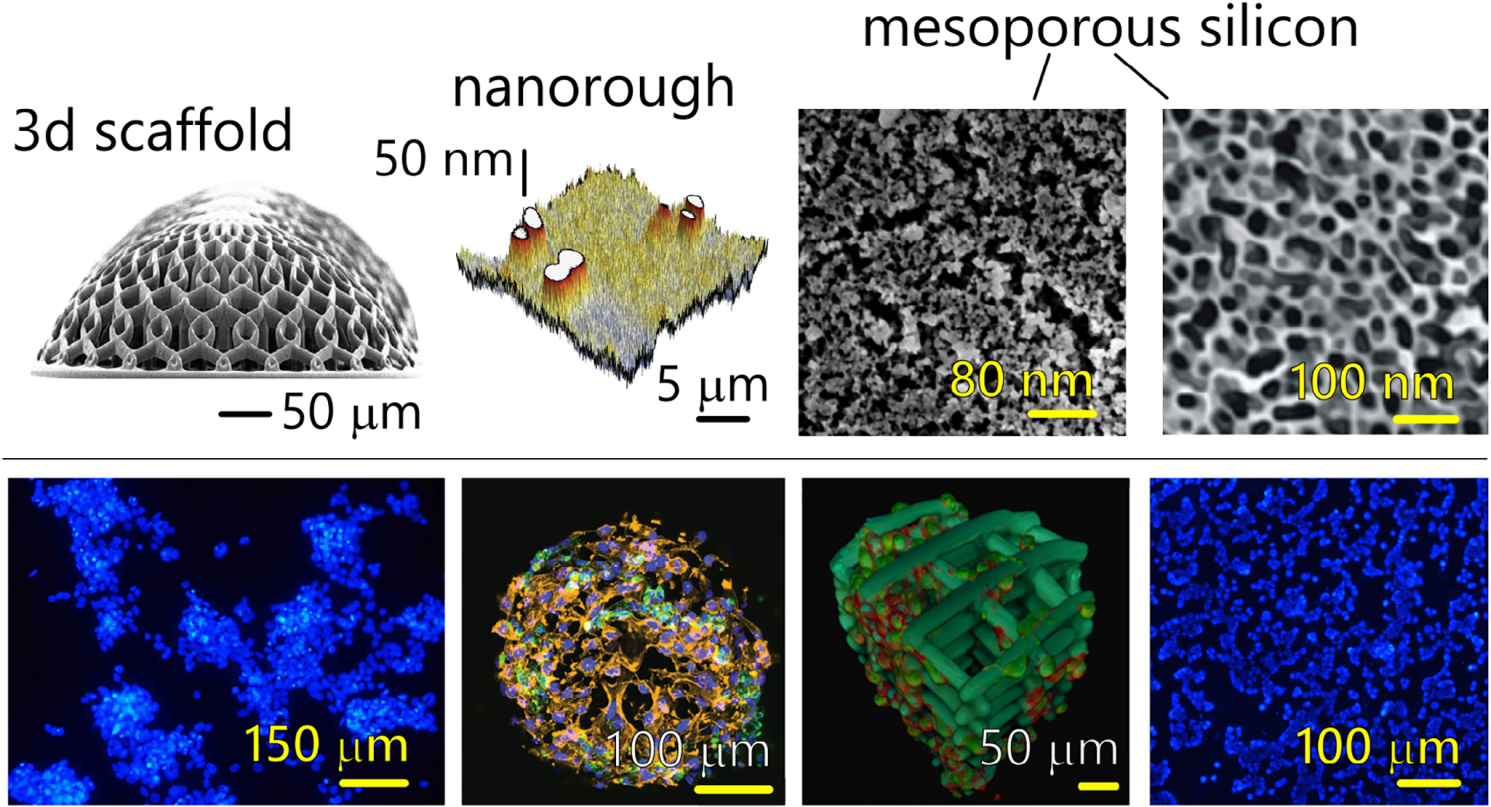
*Influence of surface nano‐topography and scaffold geometry on cancer cell network topology*. Engineered nano‐topographical surfaces and 3D scaffolds introduce controlled variations in roughness, porosity, and surface architecture, modulating cell adhesion and clustering. The biological mechanisms at the basis of nano‐topography driven cell behavior are understood, and recapitulated in a separate supporting information a). Nano‐scale surfaces and scaffolds guide the self‐assembly of cells into interconnected networks, influencing their spatial organization and functional properties b). Quantification of topological features—such as node degree, clustering coefficient, and small‐world properties—demonstrates that nano‐engineered substrates are effective driving network formation. This understanding can be possibly used in the assessment of RT by topological cell analysis: surface nano‐topography can amplify differences in cancer cell network topology post‐irradiation. This amplification effect enhances in turn the resolution of RR assessment, facilitating the identification of radio‐sensitive and radio‐resistant phenotypes with greater accuracy.

The development of cellular systems on a surface is governed by the interplay between cell‐substrate interaction forces, cell‐cell adhesion forces, and their competition. Cell adhesion to a substrate is influenced by a series of chemical and mechanical signals that begin at the bio‐interface and travel through the cell membrane to the cytoskeleton.^[^ ^51^ ^]^ Cell‐adhesion molecules (CAMs), which operate at a characteristic low nanometer scale, effectively mediate these signals.^[^ ^52^ ^]^ Consequently, cell adhesion and clustering can be adjusted by designing surface topography at the nanoscale.^[^ ^11^, ^53^ ^]^ By employing nanofabrication techniques one can create surfaces with precisely controlled nano‐topography, where roughness and *fractal dimension* are modulated independently. Fractal dimension measures surface complexity, with higher values indicating the presence of details at increasingly smaller scales.^[^ ^54^ ^]^ Among the several different techniques that exist to produce nanoscale surfaces, the simplest ones imply wet‐etching techniques^[^ ^14^, ^54^, ^55^ ^]^ and electro‐chemical etching.^[^ ^13^, ^56^ ^]^ By fine‐tuning the parameters of these fabrication processes, one can produce surfaces with specific morphological characteristics that influence cell behavior,^[^ ^11^, ^51^, ^52^, ^53^, ^57^ ^]^ (**Figure** **5**) potentially enhancing the effects of RT on cells. Previous research has shown that surfaces with intermediate roughness (20 − 40 *nm*) and high fractal dimension (> 2.4) enhance adhesion and clustering of various cell types, including human lung carcinoma,^[^ ^54^ ^]^ neuroblastoma,^[^ ^13^, ^58^ ^]^ and breast cancer cells.^[^ ^56^ ^]^ These findings have been supported by biomechanical models^[^ ^7^ ^]^ that attribute the collective behavior of cells to the combined influence of cell‐surface and cell–cell interactions.

### 2.4 3D Scaffolds for In Vitro‐Models Study of RT

2D models of cells may sometimes fail to accurately reproduce the functions of tissues and organs. While conventional clonogenic essays are based themselves on 2D set‐ups, to examine more faithfully the response of cancer cells to radiation one has to shift to 3D cell systems. Compared to 2D cultures, 3D cell models offer greater physiological relevance, more accurately mimicking tissue microenvironments, cell‐to‐cell interactions, and biological processes. 3D in vitro cancer constructs have emerged as effective tools to gain insights into cancer biology, striking a balance between simplistic 2D cultures and more intricate cancer xenografts in immunocompromised animals or syngeneic models. Recently, different 3D models have been applying to the field of radio‐oncology, showing good correlations between experimental models and actual patient's responses to RT.^[^ ^59^, ^60^, ^61^ ^]^

In particular, 3D scaffold‐based constructs provide structural support to cells and enable the investigation of cellular behavior under well‐defined culture conditions, approaching a “quasi in vivo” level. These controlled systems foster improved reproducibility and facilitate the development of more precise, scalable, high‐throughput tools, thereby producing more consistent and reliable data and saving time and costs. In 3D systems, cells preserve their spatial interactions, which are vital in determining their morphology, polarity and consequently their functions and fate. A number of studies has shown dose‐response curves, which consistently demonstrate heightened cell RR in a 3D environment compared to a 2D setting.^[^ ^62^, ^63^ ^]^

In scaffold‐based 3D models of cell cultures, cells are typically inserted in a 3D preformed structure with overall size spanning the millimeter size, but with details as small as some microns (system's resolution) and roughness in the nanometer range. Similar systems provide adequate support for cell growth enabling, among other things, the formation of cell–cell junctions, cell proliferation, the expression of specific genes and proteins: in similar systems, cells interact actively with the scaffold at the cell‐material interface. The scaffold surface can be conveniently modified to stimulate cell clustering and boost cellular response to radiation. Scaffolds can be designed using computer aided designed software, and fabricated by two‐photon polymerization following well‐assessed methods reviewed, for examples, in references.^[^ ^64^ ^]^ The surface‐characteristics of the scaffold can be conveniently tuned acting on the parameters of the process (hatching and slicing) to obtain the desired value of surface roughness at the nanoscale.

Images of the scaffold and 3D distributions of cells are adapted under the terms of the CC‐BY Creative Commons Attribution 4.0 International license (https://creativecommons.org/licenses/by/4.0):^[^ ^49^ ^]^ Copyright 2024, published by Wiley.

Images of the nano‐rough surface and of the 2D clustered distribution of cells are adapted under the terms of the CC‐BY Creative Commons Attribution 4.0 International license (https://creativecommons.org/licenses/by/4.0):^[^ ^49^ ^]^ Copyright 2017, published by Nature Portfolio.

The first mesoporous silicon surface image is adapted under the terms of the CC‐BY Creative Commons Attribution 4.0 International license (https://creativecommons.org/licenses/by/4.0):^[^ ^49^ ^]^ Copyright 2020, The Authors, published by Nature MDPI. The second mesoporous silicon surface image is adapted with permission^[^ ^58^ ^]^ Copyright 2012, published by the American Chemical Society.

### 2.5 Nanomedicine‐Based Strategies for Overcoming Cell RR

While this perspective focuses on developing a method to assess cellular RR to radiation, it is useful to briefly review the current nanotechnology and nanomedicine therapeutic approaches aimed at overcoming RR to provide context for the work. Nanomedicine has emerged as a promising approach to overcoming RR in cancer therapy by leveraging nano‐scale drug delivery systems (NDDS) and nanomaterials as radiosensitizers.^[^ ^65^, ^66^, ^67^ ^]^ These platforms enhance the therapeutic efficacy of radiotherapy, facilitating precise tumor targeting, and minimizing damage to surrounding healthy tissues. Various nano‐radiosensitizers, including gold, silver, platinum, gadolinium, and titanium‐based nanoparticles, as well as liposomes, polymeric nanocarriers, and carbon‐based nanomaterials, have been developed to enhance radiation‐induced damage at multiple biological levels.^[^ ^68^, ^69^, ^70^ ^]^ These materials exhibit excellent biocompatibility and, at the nano‐scale, can improve radiotherapy responses through their unique physicochemical, electric, and optical properties.^[^ ^71^ ^]^ The primary biological mechanisms of nanomaterial‐induced radio‐sensitization involve alterations in the cell cycle, inhibition of DNA repair, and induction of mitochondrial dysfunction.^[^ ^68^ ^]^ Functionalized nanocarriers incorporating microRNAs (miRNAs) and long non‐coding RNAs (lncRNAs) further enhance radio‐sensitization by targeting key genetic and epigenetic pathways responsible for tumor resistance. Additionally, metal nanoparticles loaded with chemotherapeutic agents enable combinatorial effects, amplifying therapeutic outcomes through enhanced targeting, biodistribution, and controlled drug release. Hybrid nanoplatforms integrating radiosensitizers, immune modulators, and chemotherapeutic drugs show promise in overcoming RR in cancers such as breast, lung, and glioblastoma. The combination of nanotechnology with molecular targeting has led to personalized and precision treatment strategies, optimizing the therapeutic index of radiotherapy while reducing systemic toxicity. Future advancements in NDDS are expected to focus on multifunctional nanoparticles that integrate imaging, targeting, and therapeutic capabilities, allowing real‐time monitoring and adaptive treatment strategies. With continued research and clinical translation, nano‐based radiosensitizers hold great potential for improving patient outcomes, reducing treatment‐related side effects, and addressing the longstanding challenge of RR in oncology.

## 3 Nano‐Topography Enhanced Topological Analysis of Cells

To improve sensitivity, enhance precision and optimize performance of topological cell analysis, we propose to integrate *networks analysis* of cancer‐cell graphs and *nanotechnology*. We aim at boosting the topological response of cells to radiation using devices modified at the nanoscale (Figure 5). Cell‐surface interactions and radiation effects can combine and direct cells to cluster into networks. Cells that are sensitive or resistant to radiation will form significantly different networks – this difference is made even larger by the underlying nanoscale geometry of the substrate. Thus, cells with low but still‐not null responsiveness to radiation (otherwise invisible to conventional analysis) can be detected thanks to the amplification effect of the substrate.

The core idea of this perspective is that both the nanoscale geometry of a surface or scaffold and radiation can influence cell clustering, and their combined effects may amplify cellular responses. As a result, this synergy can significantly enhance the sensitivity, precision, and accuracy of the method, similarly to the increased Raman signal observed in Surface‐Enhanced Raman Scattering (SERS) applications. While this perspective primarily draws from existing literature on nano‐topography‐guided cell behavior, radiation therapy, and topological cell analysis—most of which are phenomenological in nature—it remains crucial to further investigate the fundamental biological mechanisms underlying these effects. Understanding these mechanisms could reveal a common link between different drivers of cell assembly, such as nano‐topography and radiation. If elucidated, this connection could serve as a guiding principle for engineering nano‐scaffolds with enhanced efficacy for RT applications. Although further research is needed to fully explore these concepts, in a separate Supporting Information 4, we propose possible biological mechanisms underlying this work. Specifically, we first review key findings on cell‐material interactions at the nanoscale from a cell biology perspective, emphasizing recent evidence that nano‐topography can influence cell phenotype. This observation suggests that such interactions can be analyzed using epigenetics and chromatin evolution in cell nuclei. Next, we examine the biological aspects of cell‐radiation interactions in RT applications, highlighting how chromatin structure affects DNA sensitivity to radiation. We then propose that chromatin serves as the crucial link between radiation therapy and surface nano‐roughness, demonstrating how these factors can jointly drive cell behavior and clustering. Lastly, we further elaborate on the biological significance of key networks metrics, such as the small‐world coefficient.

Further to this end, much of the claims of this perspective assume that nano‐topography enhances the formation of cellular networks. In fact, this hypothesis has long been proven. The work we are submitting is part of ongoing research effort aimed at engineering bio‐interfaces and developing mathematical models^[^ ^13^, ^14^, ^15^, ^17^, ^72^, ^73^, ^74^, ^75^ ^]^ to elucidate how nano‐topography promotes cellular networking and, in turn, influences system performance. While many of these studies are discussed throughout this perspective, we provide a comprehensive overview in a separate Supporting Information 5, offering additional context, further demonstrating the feasibility and significance of this approach, and adding perspective to this work.

Thus, nano‐topography enhanced topological‐cell‐analysis can achieve accurate analysis of how cells respond to radiation therapy.

In turn, precise quantification of the sensitiveness of cells can help to choose the right dose and the right time of the treatment, increasing life expectancy of cancer patient and reducing the costs of follow‐up. Both these aspects can positively impact on the efficiency of national health services. To investigate cell‐radiation interaction, it is recommended to use both 2D and 3D models. Conventional and time modulated (i.e., FLASH RT) administration of radiation can be used to optimize the method even further and find the best treatment conditions of cancer. Moreover, different RT techniques (i.e., X‐ray, electron, proton RT) may help to demonstrate repeatability, reliability and accuracy of the method under several different working conditions. *While the use of nano‐topography to guide cell behavior is very well assessed, the integration of nanopatterned scaffolds and topological cell analysis does not have any precedent in the literature for the evaluation of the effects of radiotherapy – and offers ways to measure the performance of radiotherapy with unprecedented accuracy*.

The outcomes of a similar integration are manyfold, and can be reviewed as follows.

### 3.1 Technology Outcome

**Figure 6:**
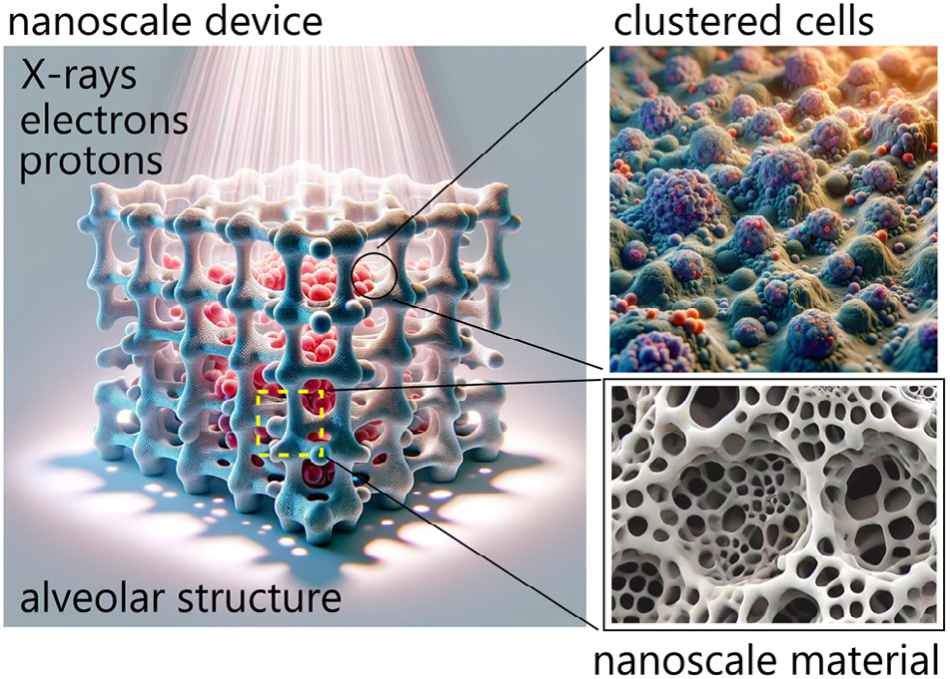
A nanoscale device is the main project outcome.

One of the main objectives of this perspective is suggesting ways for evaluating the RR and RS of cancer cells. Thus, the first product of a similar suggested approach is a nanoscale device (**Figure** **6**). If used as a substrate for cells extracted from a patient with cancer, the device will guide cells into structures with some level of complexity. Cells will be then inspected by conventional fluorescence microscopy, and resulting images examined employing the methods developed using Networks Science and AI. Convenient algorithms will give an estimate of the RS and RR of cells with elevated sensitivity and specificity, high accuracy, and low limit of detection. The RS and RR of cancer cells – assessed trough the method and the device – will be used to tailor the right dose and the right time of the treatment with RT.

### 3.2 Scientific Outcome

Alongside with the development of the device, problems that are still open will be possibly addressed. The first is elucidating the exact nature of the relationship between tissue topology, tissue physiology, and scaffold topology. While the effects of nano‐topography on cell clustering have been occasionally examined, still a systematic study on how the topology of a scaffold is correlated to the characteristics of growing cells and networks is lacking. The study herein proposed will examine how the topology, of the scaffold and of the cells, correlates to the functions of cells cultured on them. The correspondence – *equivalence* – between structure and function is a paradigm shift, in that it allows to estimate the effects of the geometry of a system on the performance of that system. Moreover, since Raman Phenotyping will be used to characterize cells, it will also be dissected whether there is a correlation between the topology of cell networks and the phenotypic distribution in those networks. This will allow establishing a correlation between scaffold topology, cell topology, and the spatial distribution of phenotypes: indicating which phenotypes are expressed by cells in response to a specific combination of parameters. Many groups worldwide are trying to develop 3D, self‐organizing, in‐vitro cell‐culture organ models. These 3D structures harbor key features of their native organs and can be used to investigate and understand biological mechanisms and diseases, including cancer. Against this background, we are setting a new powerful paradigm. The perception that the topological characteristics of a system, cell's phenotype, and the function of that system are tightly interwoven – can offer guidance to design 3D cell models and organoids with unprecedented efficiency.

### 3.3 Social outcome

This perspective centers on enhancing cancer treatment efficiency, particularly in radiotherapy. Cancer, a leading cause of morbidity and death globally, incurs substantial financial costs. RT, a crucial treatment modality, involves high expenses. This method promises to personalize treatment, potentially increasing survival rates, reducing treatment costs, and improving healthcare resource utilization. It offers a cost‐effective alternative to traditional methods, potentially revolutionizing cancer care by enabling more targeted and efficient treatment strategies. While this concept is here immediately applied to RT, it can be easily translated to other fields of biomedical research. *As for an example, the technology of prosthetic implants and medical implants*. The still‐high rejection rate of implanted devices from the human body is cause of concern of the medical industry, especially because there are no consistently effective strategies to prevent or suppress it. An effectively designed implant, guided by the principle of structure‐function equivalence, can reduce the risk of rejection and enhance performance. Further to this end, a similar principle can contribute to reduce the chance of postoperative infection, simply choosing values of geometric variables of the implant that can possibly impair the growth and proliferation of bacterial films.

## 4 Study Design and Implementation

Here we outline an experimental plan for the development and optimization of surface‐enhanced topological cell analysis. The plan is here just sketched, its exact implementation does depend on the resources, the instrumentation, and the facilities of the research groups involved in the research.

### 4.1 Fabrication

2D surfaces and 3D scaffolds with controlled geometric characteristics and nano‐topography are designed, fabricated and characterized. These platforms enable interaction of cancer cells with engineered environments.

### 4.2 Biological Preparation

Human cancer cells, including those derived from melanoma, breast, and lung cancers, provide a foundation for this research. Using conventional biological assays, one can evaluate cellular RR and RS, and build a biological database that is necessary for the rest of the study. Moreover, simple adhesion and growth of cells can be examined on 2D support and 3D devices. This approach ensures the identification of optimal conditions to enhance cellular response to radiotherapy. Selected devices will be incubated with the cells identified for this study.

### 4.3 RT Experiments

By exposing selected cell‐support systems to various RT modalities—including conventional X‐ray RT, ultra‐high dose rate FLASH electron RT, and FLASH proton RT—one can generate invaluable data on how these factors shape cellular response as a function of the supporting devices. Fluorescence microscopy can be used to acquire cellular networks on the devices and dissect the nature of cell‐cell interactions.

### 4.4 Topological Cell Analysis

Morphological and topological characteristics of cancer‐cell networks will be then examined using computational tools. Using topological data determined from networks‐science analysis, one will dissect whether and to what extent cancer traits, scaffold geometry, radiation dose, and network topology are associated. This relationship promises to unravel previously unrecognized patterns in cellular behavior.

### 4.5 Data Analysis

Once the topological characteristics of the cancer‐cell networks are determined, researchers will use statistical techniques to examine whether treatment with radiations is a special‐cause of variation of networks topology, and find specific cause‐effect (radiation‐topology) laws.

### 4.6 Artificial Intelligence

Artificial intelligence (AI), encompassing both deep learning (DL) and machine learning (ML), will serve as a powerful ally, identifying intricate relationships within extensive imaging datasets. These insights will be instrumental in predicting RR and RS across cancer cell types with increased accuracy compared to simple analytical techniques. A more detailed description on how AI can be practically implemented to examine cell response to radiation, and its integration with networks science, is reported in a separate Supporting Information 6.

### 4.7 Raman Phenotyping

At the same point, Raman spectroscopy will complement this effort by providing multifactorial characterization of 2D and 3D cultures, adding another layer of depth to our understanding of cellular phenotypes and their environmental interactions.

### 4.8 General Model of Cell Response

Finally, basing on data, a general model of correlation between scaffold‐topography, cell‐topology, and phenotypes spatial distribution will be built.

Thus, a side effect of this study‐design is understanding how the topology of cell networks and the phenotypic traits expressed by the cells correlate: it aims to establish a direct link between the physical and geometric properties of the scaffolds, the organizational patterns of cells, and their phenotypic responses. By establishing a *direct link between the physical and geometric properties of scaffolds and the functional responses of cells*, this study not only advances cancer research but also introduces a transformative framework for the design of engineered cell models and targeted therapeutic strategies.

One key ingredient in the study is *networks science*. This study proposal extends the existing knowledge on how cancer cell‐networks behave in response to RT^[^ ^49^, ^50^ ^]^ and sets a novel, powerful paradigm in radiotherapy, adding a very significant improvement with clinical implications.

## 5 Conclusion

Current RR and RS assessment of human cancer cells primarily relies on estimating the survival fraction of cells on a surface, a method with limited sensitivity, precision, and accuracy. In this perspective, we propose a multifactorial approach that considers colony shape, internal architecture, and biochemical properties. A key novelty of this approach is the integration of previously overlooked factors, such as topology, into RT assessment. Additionally, we introduce nano‐topography as an amplifying agent, enhancing the visibility of otherwise imponderable differences between radiosensitive and radioresistant cells by leveraging nanoscale materials. The introduction of materials science and nanotechnology in the field of RT, along with alternative methods of analysis based on networks science and topology, can realistically improve RT treatment of patients and, as a collateral effect, reduce costs.

Beyond RT, our approach aims to establish a broader correlation between structure and function in biological systems—a concept with far‐reaching biomedical implications. In systems like the nervous system or neuromorphic computing, function is inherently tied to structure. By measuring one, we can predict the other. The direct utilization of this principle, *from structure to function*, is useful in diagnostics, i.e., for estimating the effects of radiotherapy, the progression of cancer, the progression of a neurodegenerative disease. The indirect utilization of this principle, *from function to structure*, can be exploited in tissue engineering, reverse engineering, and related fields.

This technology has the potential to impact a wide range of fields, including cancer diagnostics and therapy, tissue repair and regeneration, neurodegenerative disease diagnosis, and neuromorphic engineering.

In the context of cancer therapy, it is essential to further elaborate on this method. The nanotechnology that we have devised is designed to assess the RR and RS of cells by analyzing their collective behaviour upon radiation exposure. This approach enables physicians to evaluate a patient's condition more accurately and design personalized treatment strategies accordingly. However, the method can be improved even further. The nanoscale material used to boost cell clustering can be, among many other possibilities, mesoporous silicon. Mesoporous silicon has the remarkable property of working as a nanoscale drug delivery system. Consequently, the method can be advanced into a theranostic system, simultaneously facilitating both diagnosis and treatment. The same platform that directs and analyzes cell assembly could also be employed to deliver radiosensitizers to targeted cells. This method could be used to study the effects of therapeutic agents on radio‐resistant cells, enabling a more sophisticated and personalized diagnostic approach for each patient. Potentially improving over the existing nanoscale drug delivery systems already employed to overcome RR and previously reviewed.

## Conflict of Interest

The authors declare no conflict of interest.

## Supporting information



Supporting Information
