# Supplementary material for: Nano‐Topography Enhanced Topological‐Cell‐Analysis in Radiation‐Therapy
Source: Adv Healthc Mater. 2025 Mar 22;14(12):2405187. doi: 10.1002/adhm.202405187 (PMC12057610; doi:10.1002/adhm.202405187)
Supplement: Supplementary file 1 — Supporting Information [file ADHM-14-0-s001.docx]

Nano-topography enhanced topological-cell-analysis in radiation-therapy

Francesca Pagliari, Maria-Francesca Spadea, Pierre Montay-Gruel,
Anggraeini Puspitasari-Kokko, Joao Seco, Luca Tirinato,
Angelo Accardo *, Francesco De Angelis *, Francesco Gentile *

**Supporting Information**

**Supporting Information 1**. *On the influence of 2D nanoscale surfaces on cell network topology, and comparison with 3D scaffolds*.

There is a number of reported examples that describe how the geometry of 2D surfaces influence cell network topology. In reference ^[1]^, results from an experimental campaign illustrate that neuroblastoma N2A cells on porous silicon chips with a small pore size and large fractal dimensions up to Df~2.8 form highly clustered, small world neuronal cell networks. In reference ^[2]^, it is reported that neuronal cells on nanoscale surfaces assemble into unsymmetrical patterns, in proportion to the roughness of the surface in the low nanometer range, and that this characteristic network topology improves the overall information transported in the system. In a similar work ^[3]^, it is shown that neuronal cells on zinc oxide nanowire surfaces create supracellular structures with an upper-bounded cluster size – resembling the architecture of the cortical mini-columns, i.e. the fundamental chips of the brain. In reference ^[4]^, researchers explored the influence of surface elasticity in the low MPa range, on the behavior of primary hippocampal neural cultures. They found that the larger the compliance of the surface, the higher the activity of neuronal cells, in terms of quantity, quality and density of action potentials. Further to this end, a recently published work ^[5]^ reviewed how surface topographies of cell culture substrates can be used to generate in-vitro cell culture environments, and replicate the aspects, characteristics, and functions typical of in vivo cell niches. In reference ^[6]^, Bhuvanesh and colleagues examined the dynamic properties of 2D laminin material, such as self-renewal and self-healing, highlighting their importance for the maintenance of tissues and organs.

Thus, nanoscale 2D surfaces serve as the crucial link between traditional 2D cell culture models and more advanced 3D templates. They provide the necessary complexity for cell colony growth while enhancing physiological relevance, drug response accuracy, and predictive power—hallmarks of 3D cell models ^[7]^. At the same time, they retain the simplicity and cost-effectiveness of conventional 2D cultures, making them an optimal bridge between these two approaches. However, the superiority of 3D cell culture models over traditional 2D cultures is widely recognized.

Three-dimensional (3D) cell culture models have emerged as a superior alternative to traditional two-dimensional (2D) cell cultures due to their ability to more accurately replicate the complex environment of living tissues. Unlike 2D cultures, where cells grow in flat monolayers and lack meaningful interactions with their surroundings, 3D cultures allow cells to communicate with each other and with the extracellular matrix in a manner that closely mirrors in vivo conditions. This structural and physiological similarity makes 3D cultures an invaluable tool in biomedical research.

One of the most significant advantages of 3D cell cultures lies in their ability to provide more accurate drug response data. In conventional 2D cultures, all cells are uniformly exposed to nutrients and therapeutic agents, which does not accurately reflect the reality within human tissues, where gradients of oxygen and nutrients exist. In contrast, 3D cultures better mimic the microenvironment found in the human body, leading to more reliable drug metabolism studies. For instance, tumor cells grown in 3D often exhibit drug resistance and stem-cell-like behavior, characteristics that are crucial in understanding how cancer therapies will perform in actual patients. This makes 3D cultures particularly useful for drug discovery and development, as they provide more predictive insights into efficacy and toxicity compared to traditional models.

Beyond drug testing, 3D cultures also enhance the study of cellular behavior and interactions. In a 2D environment, cells tend to proliferate at an unnaturally rapid pace, as they are not constrained by the physical barriers and limitations present in actual tissues. In contrast, 3D cultures introduce natural constraints, allowing researchers to study realistic growth rates and responses to external stimuli. Additionally, because these models permit the formation of complex cellular junctions, they facilitate more accurate gene expression patterns and protein synthesis, offering a closer approximation of how cells behave in their natural state.

A key benefit of 3D cell culture technology is its potential to reduce reliance on animal models. Given the ethical and financial challenges associated with animal testing, the ability to create organoids and tumor-on-a-chip models provides an appealing alternative for studying disease progression and treatment effects. These models, derived from patient cells, also open the door for personalized medicine, allowing clinicians to test therapies on a patient-specific basis before actual treatment begins.

Moreover, 3D cultures have proven invaluable in metabolic research, enabling scientists to study metabolic cooperation between different cell types with a level of accuracy that 2D cultures cannot achieve. By simulating the depth and complexity of real tissues, they offer new perspectives on how drugs, nutrients, and cellular byproducts interact within the human body.

Despite these advantages, 2D cultures remain in widespread use due to their simplicity and cost-effectiveness. However, as biomedical research progresses, the transition towards 3D models is becoming increasingly necessary. Their ability to replicate human tissues more faithfully not only improves the accuracy of experimental results but also has the potential to accelerate advancements in cancer research, regenerative medicine, and drug development. With continuous improvements in 3D culture techniques, they are poised to bridge the gap between traditional in vitro studies and in vivo applications, ultimately paving the way for more effective and personalized medical treatments.

**Supporting Information 2**. *Correlation between the dose of ionizing radiation and the topology of resulting cancer cell networks*.

The correlation between the dose of ionizing radiation and the topology of cancer cell lines has been explored in preliminary research studies ^8,9^. Both studies provide evidence that radiation-induced changes in cancer cell networks follow predictable topological patterns, with increasing doses leading to fragmented and more heterogeneous cellular distributions.

*A first study*, entitled "Human Cancer Cell Radiation Response Investigated through Topological Analysis of 2D Cell Networks" ^[8]^, analyzed four different cancer cell lines: H4 (epithelial neuroglioma), H460 (lung cancer), PC3 (bone metastasis of prostate cancer), and T24 (urinary bladder cancer). These cell lines were exposed to radiation doses ranging from 0 to 6 Gy, and their topological changes were monitored. The key finding of this study was that as the radiation dose increased, the small-world coefficient (sw) also increased, while the characteristic path length (cpl) and graph degree (k) decreased. This pattern indicated that higher doses disrupted the uniformity of the cellular networks, leading to fragmented, irregularly clustered cell distributions. In particular, T24 cells exhibited the greatest sensitivity to radiation, showing the most pronounced network disintegration, followed by H4, H460, and PC3 cells. Notably, when the radiation dose reached 4 Gy or higher, the small-world coefficient exceeded 1 ($sw>1$), a strong indication of disrupted connectivity and increased heterogeneity in the cell distribution.

*The second study*, "Human Lung-Cancer-Cell Radioresistance Investigated through 2D Network Topology" ^[9]^, focused exclusively on lung cancer cell lines, analyzing H460, A549, and Calu-1 cells subjected to radiation doses up to 8 Gy. Like the previous study, this research utilized network-based metrics to assess changes in cell connectivity and clustering under radiation stress. The results revealed distinct differences in radioresistance among the lung cancer cell lines. The H460 cell line exhibited the highest sensitivity, with the small-world coefficient increasing nearly fourfold at 8 Gy, indicating substantial network fragmentation and a loss of structured cellular organization. In contrast, A549 cells displayed moderate sensitivity, while Calu-1 cells were the most radioresistant, showing only minimal changes in their network topology, even at the highest radiation dose. A particularly notable observation was that higher radiation doses led to increased discontinuity in cell clusters, with fewer interconnections between surviving cells. The study also found that at doses above 6 Gy, the characteristic path length (cpl) dropped sharply, meaning that while cells still formed networks, they became more tightly packed in smaller, less connected clusters.

Despite differences in the cancer types and dose ranges studied, both investigations identified common trends in the topological responses of cancer cells to radiation. Specifically: (i) Higher radiation doses led to an increase in the small-world coefficient (sw), with values exceeding 1 ($sw>1$) at critical doses (4–6 Gy in multi-cancer cells, 6–8 Gy in lung cancer cells). (ii) Clustering coefficient (cc) remained relatively stable or increased slightly, suggesting that, despite network fragmentation, localized cell groups remained interconnected. (iii) Characteristic path length (cpl) consistently decreased with dose, indicating that while global connectivity was disrupted, surviving cells tended to form closer clusters. (iv) Graph degree (k) declined across all cell lines, reflecting a reduction in the number of direct cell-cell interactions as radiation dose increased.

**Supporting Information 3**. *Sensitivity of topological cell analysis and thorough examination of the limits of clonogenic assays*.

While this paper was not focused on quantifying the performance of *topological cell analysis* and it was more a proof of existence, it is however useful to try to estimate the sensitivity of the method, and compare it to the sensitivity of classic clonogenic assays, to position this perspective in context. In the following of this section, we will better describe how clonogenic assays work, especially pertaining to tissue culture plastic (TCP) models – i.e. still the gold standard for determining the radio-sensitivity of cells ①. We will then critically comment on the critical aspects of clonogenic assays, we will examine its robustness and inter-assay precision, and will expound on its limitations ②. Then, we will provide a quantitative estimate of the sensitivity of clonogenic assays, and compare it to that of topological cell analysis –for the cases reported in literature thus far ③. Lastly, we will recapitulate the main results of this comparative analysis ④.

**SI 3.1** *Tissue culture plastic models in clonogenic assays* ①. Clonogenic assays are a fundamental method used to evaluate the ability of a single cell to survive, proliferate, and form a colony following exposure to ionizing radiation. In these assays, cells are seeded at a low density on a culture substrate, exposed to varying radiation doses, and then incubated for a period (typically 1–3 weeks) to allow for colony formation. Colonies consisting of at least 50 cells are then stained and counted to determine the surviving fraction. This method provides a direct measure of reproductive cell survival and is widely used to assess the effectiveness of radiotherapy and other cytotoxic treatments. TCP models form the traditional basis for clonogenic assays, utilizing polystyrene or polycarbonate culture dishes, flasks, and well plates as cell substrates. These surfaces are often coated with biochemical layers, such as laminin, fibronectin, or collagen, to enhance cell adhesion. TCPs are inexpensive, sterilizable, and highly reproducible, making them an attractive platform for high-throughput radiobiological experiments. The rigid nature of TCP materials facilitates easy imaging and colony counting, allowing for systematic assessment of radiation-induced cellular damage and repair. Classical clonogenic assays in TCP models remain a cornerstone in radiobiology ^[10]^, offering a simple, cost-effective, and highly reproducible method to assess cell survival following radiation exposure. However, their major limitations stem from the artificial 2D environment, which does not accurately reflect the 3D tumor microenvironment. This can lead to altered cell adhesion properties, an overestimation of radiation-induced damage, and a lack of physiological tumor heterogeneity. While TCP-based assays continue to be valuable, emerging 3D culture systems, such as tumor spheroids, organoids, and scaffold-based models, offer more physiologically relevant alternatives for studying radiotherapy effects in a more biomimetic context.

A more detailed and comprehensive discussion of classic clonogenic assays is provided below.

*Fundamentals of Classical Clonogenic Assays.* Clonogenic assays, also known as colony formation assays, are a well-established technique used to assess the ability of a single cell to survive, proliferate, and form a colony after exposure to a cytotoxic agent, such as ionizing radiation. The fundamental principle of this assay is to determine the reproductive capacity of cells post-treatment by measuring their ability to form large, viable colonies. A colony is typically defined as a cluster of at least 50 cells derived from a single progenitor.

The classical clonogenic assay involves several key steps: (1) cell Seeding: Cells are plated at a low density in tissue culture plastic (TCP) flasks, Petri dishes, or well plates to ensure that individual cells have sufficient space to grow into distinct colonies. (2) Radiation Exposure: Cells are subjected to various doses of ionizing radiation, such as X-rays, protons, or electron beams, to evaluate their survival and proliferative potential under different conditions. (3) Colony Growth: Following irradiation, cells are incubated for a period of 7–21 days, allowing them to proliferate and form colonies. (4) Fixation and Staining: Colonies are fixed using methanol or ethanol and stained with dyes such as crystal violet or methylene blue to enhance visibility. (5) Colony Counting and Analysis: Colonies consisting of at least 50 cells are counted manually or using automated imaging systems. The surviving fraction is then calculated by normalizing colony counts to those in unirradiated control samples.

Clonogenic assays provide a direct measure of cell survival and proliferation, making them an essential tool in radiobiology for quantifying the effectiveness of radiation therapy and determining the intrinsic radiosensitivity of different cell types.

*Advantages of Classical Clonogenic Assays in TCP Models.* Classical clonogenic assays using TCP-based models offer several advantages that make them the gold standard for cell survival studies in radiobiology: (1) High Reproducibility and Standardization: TCP models, typically made of polystyrene or polycarbonate, provide a consistent and controlled environment for cell growth. Their widespread use ensures comparability across studies. (2) Ease of Use and Cost-Effectiveness: TCP-based clonogenic assays are relatively simple to perform, requiring minimal specialized equipment. Their low cost makes them accessible for high-throughput experiments. (3) High Throughput Capability: TCP models can be used in multi-well plate formats, allowing simultaneous testing of multiple conditions, including different radiation doses and combination therapies. (4) Established Protocols and Extensive Literature: Decades of research using TCP-based clonogenic assays provide a well-documented methodological framework, enabling researchers to compare results with previously published data. (5) Quantitative and Functional Assessment: The assay not only determines the survival fraction but also provides insights into cell proliferation, DNA damage repair mechanisms, and long-term effects of radiation.

*Limitations of Classical Clonogenic Assays in TCP Models.* Despite their advantages, TCP-based clonogenic assays have significant limitations that impact their physiological relevance in radiobiological studies: (1) Lack of Three-Dimensional (3D) Structure: TCP models impose a rigid, two-dimensional (2D) environment, which fails to replicate the native tumor microenvironment (TME). In vivo, cancer cells exist in a complex 3D architecture with extracellular matrix (ECM) interactions, oxygen gradients, and variable nutrient supply. This limitation affects cell behavior and treatment response. (2) Altered Cell Adhesion and Mechanotransduction: The stiffness of TCP materials (Young’s modulus of $\sim2-4 GPa$) is significantly higher than that of soft tissues (which range from a few pascals to kilopascals). This discrepancy influences cell adhesion, integrin signaling, and mechanotransduction pathways, ultimately impacting proliferation and radiosensitivity. (3) Overestimation of Radiation-Induced DNA Damage: Cells in TCP-based models are uniformly exposed to oxygen and nutrients, whereas in vivo tumors exhibit hypoxic regions that alter DNA damage repair mechanisms and radiation sensitivity. Consequently, TCP models may overestimate radiation-induced damage and apoptosis compared to hypoxic, 3D tumor environments. (4) Absence of Tumor Heterogeneity and Cell-Cell Interactions: In vivo tumors contain heterogeneous cell populations, including stromal and immune cells, which influence tumor response to radiation. TCP-based clonogenic assays lack this complexity, leading to potential discrepancies between in vitro and clinical outcomes. (5) Limited Relevance for Personalized Medicine: While TCP models provide a standardized platform, they do not accurately capture patient-specific variations in tumor architecture, ECM composition, and cellular heterogeneity. This limits their translational value in predicting patient responses to radiotherapy. (6) Potential Artifacts in Dose Distribution: TCP materials may interact with certain radiation types, particularly electron beams, causing unintended variations in dose distribution. This can introduce inconsistencies in clonogenic survival results when translating findings to in vivo settings.

*Conclusions.* Classical clonogenic assays in TCP models remain a cornerstone in radiobiology, offering a simple, cost-effective, and highly reproducible method to assess cell survival following radiation exposure. However, their major limitations stem from the artificial 2D environment, which does not accurately reflect the 3D tumor microenvironment. This can lead to altered cell adhesion properties, an overestimation of radiation-induced damage, and a lack of physiological tumor heterogeneity. While TCP-based assays continue to be valuable, emerging 3D culture systems, such as tumor spheroids, organoids, and scaffold-based models, offer more physiologically relevant alternatives for studying radiotherapy effects in a more biomimetic context.

**SI 3.2** *Critical aspects of clonogenic assays* ②. Clonogenic assays remain a cornerstone in assessing radiosensitivity in cancer research, but their *robustness and inter-assay precision* have been subjects of scrutiny. Studies indicate that while the method is widely used and considered the gold standard, several critical limitations impact its reliability.

*Robustness and Inter-Assay Precision.* The robustness of clonogenic assays is often challenged by variability in experimental conditions, including differences in plating efficiency, cell density, assay volume, incubation time, and cell cooperation effects ^[11]^. While inter-assay precision for key survival endpoints such as SF2 (surviving fraction after 2 Gy) and D10 (dose that reduces survival to $10\%$) falls within an acceptable variance $(<30\%$), yet this figure cannot be improver further to the previously cited issues. Automated imaging and time-resolved methodologies have been suggested to enhance precision by tracking colony growth more dynamically rather than relying on fixed size thresholds ^[12]^.

*Limitations and Challenges.* One fundamental limitation of clonogenic assays is the assumption of a linear correlation between seeded cells and resulting colonies, which does not hold for all cell lines due to cellular cooperation effects ^[11]^. This phenomenon results in non-linear growth behavior, undermining plating efficiency-based survival calculations. Additionally, the reproducibility of clonogenic assays is compromised by inconsistencies in reporting methodologies, with many studies failing to document critical experimental parameters, such as the number of biological and technical replicates, radiation sources, and dose rates ^[13]^. This lack of standardization significantly impacts data comparability across different research groups. Furthermore, the time-consuming and labor-intensive nature of clonogenic assays, along with their sensitivity to operator-dependent variability, poses practical challenges for high-throughput applications ^[14]^.

*Thus,* while clonogenic assays provide valuable insights into radiosensitivity, their reliability is hindered by methodological inconsistencies, cell-line-specific behaviors, and limitations in plating efficiency-based calculations. Addressing these issues through improved standardization, automation, and alternative mathematical models could enhance their robustness and precision, making them more suitable for modern radiobiological research ^[13]^.

**SI 3.3** *An estimate of the sensitivity of clonogenic assays, and comparison with topological cell analysis* ③*.* Colony formation assays rely upon the estimation of the surviving fraction (SF) of cells, i.e. the number of colonies that arise after treatment of cells ^[15]^. Such a figure is given by the formula

| $\mathrm{SF}=\frac{no. of colonies formed after treatment}{no. of cells seeded \times\mathrm{PE}}$ | (3.1) |
| --- | --- |

Where PE, in turn, is the plating efficiency, i.e. the number of colonies to the number of cultured cells in absence of external treatment, i.e. the control. PE is a biologic characteristic of the specific cell line under analysis. SF varies with the radiation dose (D). Such a variation is typically described by a linear quadratic (LQ) model ^[15]^:

| $\mathrm{SF}=exp\left( -\alpha D-\beta D^{2} \right)$ | (3.2) |
| --- | --- |

Where $\alpha$ and $\beta$ are model parameters tuned by experiments. The sensitivity $s$, i.e. the change of output per change of input of the SF function, is readily determined from Equation (3.2) as:

| $s={\partial SF}/{\partial D}=-\left( \alpha+2\beta D \right) exp\left( -\alpha D-\beta D^{2} \right).$ | (3.3) |
| --- | --- |

**Supporting Information Figures 3.1** to **3.3** report values of SF and sensitivity relative to different cell lines, namely H460 lung cancer cells (**Supporting Information Figure 3.1**), PC3 prostate cancer cells (**Supporting Information Figure 3.2**), T24 human urinary bladder cancer cells (**Supporting Information Figure 3.3**). For these, results of clonogenic assays were obtained from the literature ^14,16^. Notice that, in the diagrams, values of survival fraction are expressed in non-dimensional units. Thus, the corresponding values of sensitivity are given in 1/Gy units: indicating the fraction of colonies that are removed from the plate for unit of radiation dose exposure. The absolute values of sensitivity determined by clonogenic assays for these cell lines vary in the $0-0.1$ interval for H460 cells, in the $0-0.2$ interval for PC3 cells, in the $0-0.4$ interval for T24 cells. For the H460 and PC3 cell lines, the sensitivity to the treatment is maximum for low ($\sim0 Gy$) and high ($\sim6 Gy$) values of external applied dose, and less relevant for intermediate values of radiation dose in the $2-4$ Gy interval. For the T24 cells, the absolute value of sensitivity steadily decreases for increasing values of radiation dose.

In contrast, the diagrams in the **Supporting Information Figure 3.4** display the small-world coefficients—a topological measure of networks—determined for the same cell lines, as reported in a recently published study ^[8]^. In this case, sensitivity is expressed as the change in the small-world coefficient relative to the change in radiation dose. However, since the small-world coefficient is *dimensionless* by definition, sensitivity is also expressed in units of 1/Gy. Thus, the sensitivity of topological cell analysis is directly comparable to that of classic clonogenic assays.

Values of sensitivity resulting from topological cell analysis fall in the $0-0.12$ interval for H460 cells, in the $0-0.35$ interval for PC3 cells, in the $0-1.1$ interval for T24 cells. These values are either equivalent to or exceed the values of sensitivity obtained from clonogenic assays for all considered cell lines. The observed increased sensitivity of simple topological cell analysis over conventional clonogenic assays - for the considered cases – suggests that the introduction of nano-topography could further improve the assessment of cancer cell responses to radiation, enhancing overall performance.

**SI 3.4** *Conclusions* ④*.* Analysis of the existing body of literature and a comparative study to the recently introduced topological cell analysis, suggest that conventional clonogenic assays suffer from limitations that hamper its efficacy. The main points of concern have been identified from the Authors of this perspective as follows.

a. *Time*. The time necessary for conventional clonogenic assays ranges from 1 to 3 weeks, compared to the few days required for topological cell analysis.

b. *Inter-colony versus intra-colony variability*. Conventional clonogenic assays focus on the number of colonies evolving on a cell-culture plate upon exposition to radiation. In contrast, topological cell analysis examines the internal structure of individual colonies, providing access to a greater quantity and quality of information. This enhanced level of detail translates into greater sensitivity compared to conventional clonogenic assays. The integration of nano-topography can improve the method even further.

c. *Resolution*. Further to this point, clonogenic assays are based on the calculus of the SF variable, that in in turn depends on the number ($N$) of colonies on a plate (eq. 3.1). Depending on the cell line, radiation dose, time of the analysis, and the cells initially seeded, N can be lower than $100$: in some cases as low as $30-40$ ^[15]^. As a result, under these conditions, the minimum detectable variation of SF is larger than $2$ to $3$ non-dimensional units ($2-3 \%$), leading to poor resolution.

d. *Robustness and reliability*. Robustness of clonogenic assays is often challenged by variability in experimental conditions, including differences in plating efficiency, cell density, assay volume, incubation time, and cell cooperation effects.


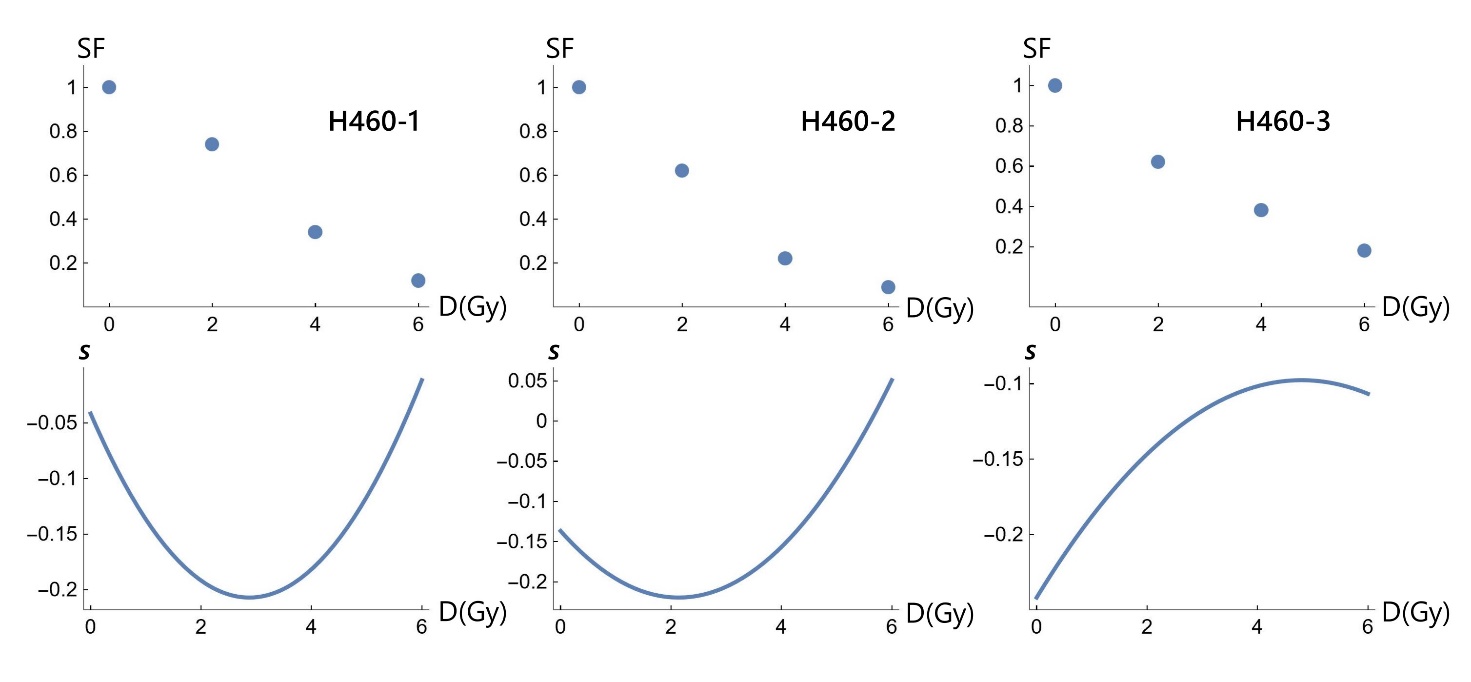


**Supporting Information Figure 3.1**


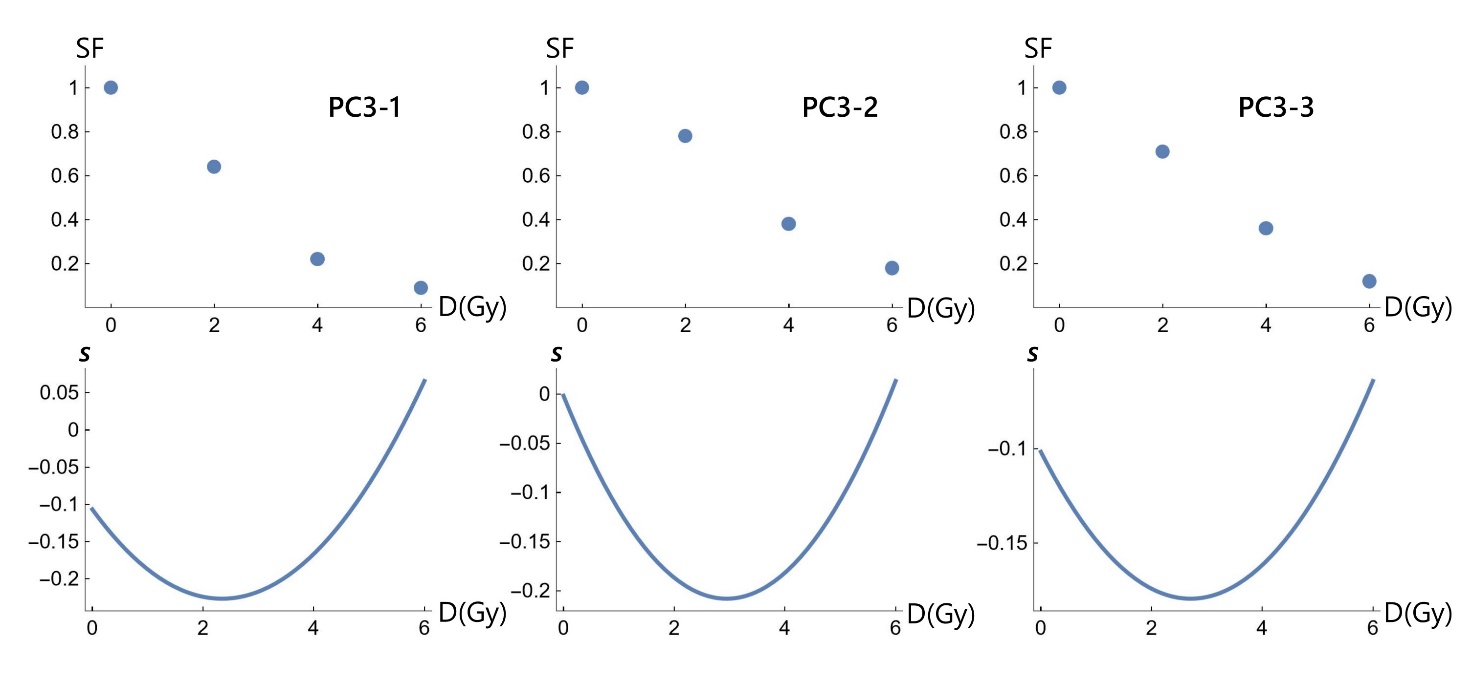


**Supporting Information Figure 3.2**


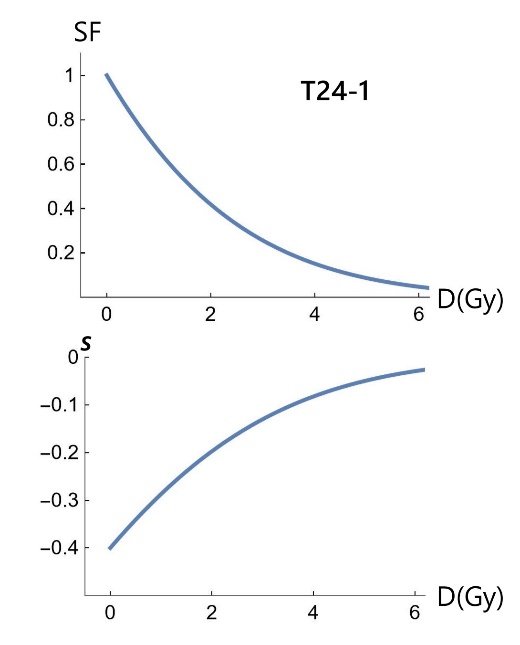


**Supporting Information Figure 3.3**


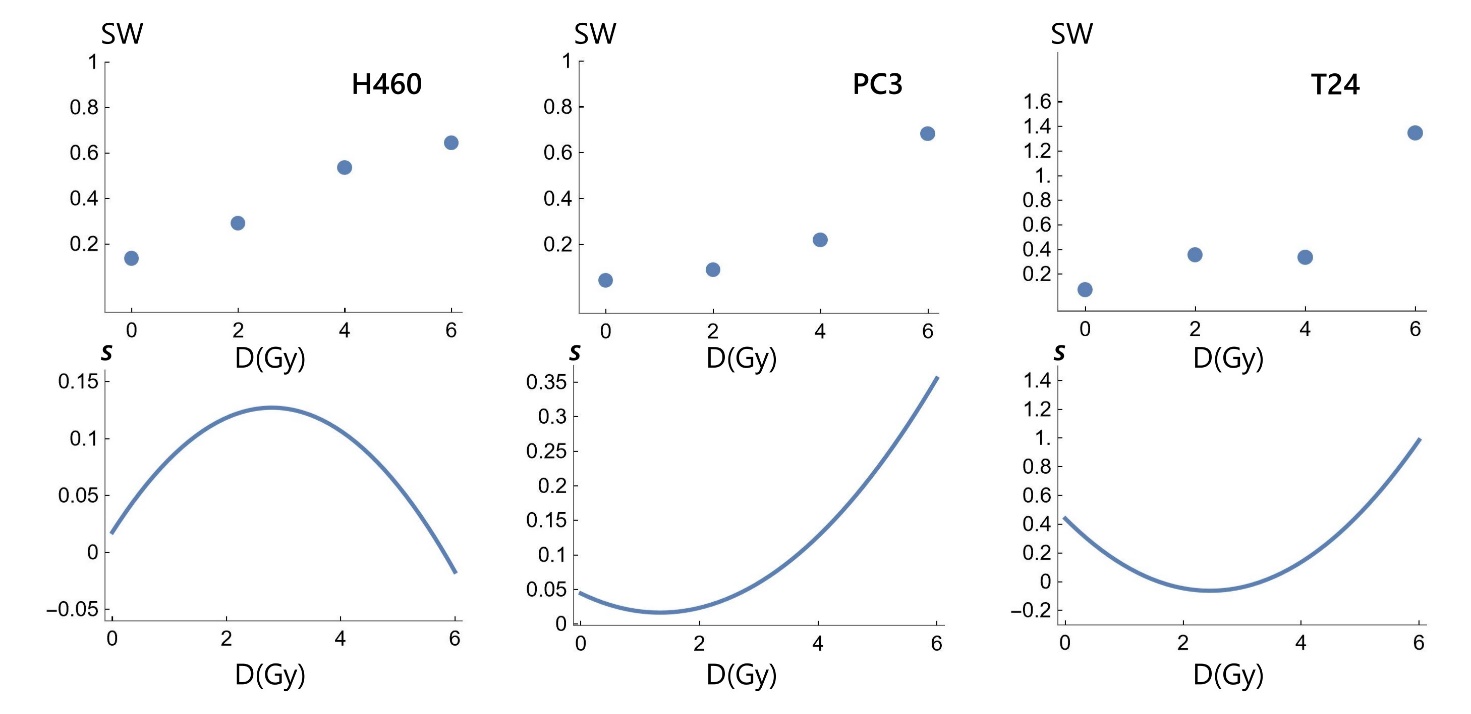


**Supporting Information Figure 3.4**

**Supporting Information 4**. *Biological mechanisms of nano-topography and radiation as special cause of cell-assembly and clustering*.

While this perspective primarily draws from existing literature on nano-topography-guided cell behavior, radiation therapy, and topological cell analysis—most of which are phenomenological in nature—it is nonetheless valuable to further explore the fundamental biological mechanisms underlying these effects, as suggested by the Reviewer. We will organize this section as follows.

First, we review the main results pertaining cell-material interaction at the nanoscale, from a cell-biology perspective ①. In doing so, we stress the recently observed evidence that nano-topography influences the phenotype of cells, and can be therefore analyzed using epigenetics and the evolution of chromatin in cell nuclei ②. Then, we examine the biological aspects of cell-radiation interaction in RT applications, evidencing how the structure of chromatin influences the sensitivity of DNA to radiation ③. Then, we will that the chromatin is the link between radiation therapy and surface nano-roughness, and thus these elements can combine in driving cell behavior and clustering ④. Lastly, we elaborate on the biological significance of key networks metrics, such as the small-world coefficient ⑤.

**SI 4.1** *Biological mechanisms of cell-material interaction* ①*.* The fundamental biological mechanisms that regulate cell behavior upon interaction with nanoscale surfaces are primarily governed by the extracellular matrix (ECM), integrin-mediated adhesion, and mechano-transduction pathways ^17,18^. When cells encounter nanoscale topographies, they sense the surface features through integrins, which are transmembrane receptors that bridge the ECM with the cytoskeleton ^[19]^. These interactions influence protein adsorption, which dictates the availability and conformation of ECM proteins such as fibronectin, vitronectin, and collagen ^[17]^. Upon ligand binding, integrins cluster and form focal adhesions, which serve as mechanosensitive hubs that regulate intracellular signaling pathways, including the activation of focal adhesion kinase (FAK), Rho-GTPases, and the Hippo signaling cascade ^[20]^. These pathways modulate cytoskeletal dynamics, cell proliferation, differentiation, migration, and apoptosis ^[19]^. Furthermore, nano-topography can work in combination with mechanical cues, such as substrate stiffness and curvature, affecting how forces are transmitted across the cell membrane and leading to adaptive cellular responses ^[20]^. Collectively, these nanoscale interactions orchestrate cell fate and behavior in tissue development, regeneration, and biomaterial integration.

**SI 4.2** *Epigenetics and chromatin as indicators of nano-topography induced cell behavior* ②*.* Thus, review of the existing body of literature suggests that nano-topography significantly influences cell phenotype by regulating cell adhesion, cytoskeletal organization, and gene expression. Cell *phenotype* refers to the observable characteristics and functional traits of a cell, including its morphology, proliferation rate, differentiation state, and metabolic activity. These traits are determined by the cell’s genetic information, environmental interactions, and regulatory mechanisms. *Epigenetics*, on the other hand, is the study of heritable but reversible changes in gene expression that do not involve alterations in the DNA sequence. These changes occur through mechanisms such as DNA methylation, histone modifications, and non-coding RNA interactions, which regulate chromatin structure and gene accessibility.

Epigenetic modifications play a crucial role in mediating nano-topography-induced phenotypic changes. One key mechanism involves histone modifications and chromatin remodeling, which regulate gene accessibility and transcription. For example, studies on osteoblasts cultured on nanostructured titanium surfaces show that these surfaces modulate the expression of long non-coding RNAs (lncRNAs), which in turn regulate osteogenic differentiation by interacting with key bone-related genes ^[21]^. Additionally, histone methylation and acetylation are influenced by nano-topography, affecting transcriptional activity in response to mechanical cues ^[22]^. DNA methylation patterns also shift, leading to stable alterations in gene expression without changing the underlying genetic code ^[23]^. Furthermore, non-coding RNAs, such as microRNAs and lncRNAs, contribute to nano-topography-driven epigenetic changes. These molecules interact with chromatin-modifying enzymes and transcription factors, regulating key pathways involved in cell fate decisions ^[22]^. In stem cells, nano-topography has been shown to enhance osteogenic differentiation by modulating chromatin accessibility, increasing the expression of osteogenic markers while repressing genes related to other lineages ^[22]^.

In summary, nano-topography exerts profound effects on cell phenotype by triggering mechano-transduction pathways that lead to epigenetic reprogramming. Through chromatin remodeling, histone modifications, and non-coding RNA interactions, cells can "remember" and sustain the effects of their physical environment, ultimately influencing their differentiation and functional behavior ^21,22^.

**SI 4.3** *The structure of chromatin influences the sensitivity of DNA to radiation* ③*.* In radiation therapy (RT), the biological response of cells to radiation is highly dependent on the structure of chromatin, which plays a crucial role in determining DNA sensitivity to radiation-induced damage. Chromatin exists in two primary forms: tightly packed heterochromatin and more relaxed euchromatin. Studies show that euchromatin, which is more open and transcriptionally active, is significantly more susceptible to radiation-induced DNA double-strand breaks (DSBs) compared to condensed heterochromatin ^[24]^. This increased sensitivity arises because euchromatin's loose structure makes DNA more accessible to ionizing radiation and reactive oxygen species, increasing the likelihood of strand breaks.

Conversely, heterochromatin is more resistant to radiation-induced damage due to its dense packing and the presence of chromatin-associated proteins, which provide a protective shield against direct radiation effects ^[24]^. However, when DSBs do occur in heterochromatin, they are repaired more slowly compared to those in euchromatin, as the repair machinery requires chromatin remodeling to access the damage sites ^[25]^. This delayed repair can lead to persistent DNA damage and contribute to genomic instability, which is relevant for tumor cells undergoing radiation therapy.

Radiation exposure also induces epigenetic changes, including chromatin remodeling, histone modifications, and alterations in gene expression, which influence the cellular response to radiation damage ^[26]^. Some tumors develop resistance to radiation through adaptive chromatin modifications that enhance DNA repair mechanisms or alter gene expression to promote survival ^[26]^. Understanding the interplay between chromatin structure and radiation sensitivity is critical for optimizing RT strategies, as targeting chromatin remodeling factors could enhance radiosensitivity in resistant tumors, improving therapeutic outcomes ^[26]^.

**SI 4.4** *Chromatin as a link between RT and surface nano-roughness in determining cell behavior and clustering* ④*.* Chromatin structure may serve as a critical link between nano-topography and radiation therapy (RT) in shaping cell behavior and response. Nano-topography influences chromatin organization through mechano-transduction and epigenetic modifications, altering gene expression and cellular phenotype ^21,22^. Similarly, RT sensitivity is determined by chromatin state, with open euchromatin being more susceptible to DNA damage, while compact heterochromatin resists radiation-induced breaks but exhibits slower repair ^24,25^. This shared chromatin-dependent regulation suggests that nanoscale surface cues could precondition cells for enhanced or reduced RT sensitivity by modulating chromatin accessibility and repair efficiency. Understanding this interplay could lead to novel therapeutic strategies where nano-engineered surfaces are used to manipulate chromatin states, optimizing RT efficacy and cell clustering dynamics in tumor treatment ^[26]^.

**SI 4.5** *On the biological significance of key networks metrics* ⑤*.* Key network metrics, such as the clustering coefficient and the small-world coefficient, play a crucial role in understanding the organization and functionality of biological systems. The clustering coefficient measures the tendency of nodes in a network to form tightly interconnected groups, which is essential in biological contexts such as protein interaction networks, metabolic pathways, and gene regulatory networks, where functional modules emerge from highly clustered regions ^27,28^. A high clustering coefficient suggests a robust system where localized interactions are efficient and resilient to perturbations, promoting functional specialization and redundancy in biological networks ^[29]^. The small-world coefficient, on the other hand, reflects a balance between local clustering and global connectivity, ensuring that biological networks maintain short path lengths for rapid communication while preserving modularity ^[30]^. This property is evident in brain connectivity, where a small-world topology enables efficient information transfer with minimal energy costs, and in cellular networks, where signaling pathways integrate diverse stimuli with high efficiency ^[31]^. These metrics collectively highlight how biological networks optimize information flow, robustness, and adaptability, underpinning essential physiological and developmental processes.

**Supporting Information 5**. *Review of the ongoing research effort to prove the assumption that nano-topography enhances the formation of cellular networks*.

The perspective work is part of a long-standing research effort focused on engineering bio-interfaces and developing mathematical models to understand how nano-topography enhances cellular networking and, in turn, influences system performance. While several of these studies are already cited in the study, however, for completeness, we do provide here a detailed commentary on these articles, further demonstrating the feasibility and significance of *nano-topography enhanced topological cell analysis*.

In the 2010 study "Cells preferentially grow on rough substrates" published in *Biomaterials* ^[32]^, Gentile et al. investigated how substrate nano-topography influences cell adhesion and proliferation. They found that cells exhibit a preference for growing on rough surfaces, which has significant implications for the design of bio-adhesives and tissue engineering applications.

Marinaro et al., in their 2015 paper "Networks of neuroblastoma cells on porous silicon substrates reveal a small world topology" in *Integrative Biology* ^[1]^, fabricated porous silicon chips with varying pore sizes and fractal dimensions. They observed that neuroblastoma N2A cells adhered more firmly and proliferated better on these porous surfaces compared to flat silicon substrates. Notably, the cells formed highly clustered networks exhibiting small-world topology, suggesting enhanced efficiency in information processing within such architectures.

In the 2017 *Scientific Reports* article "Nano-topography Enhances Communication in Neural Cells Networks," Onesto et al. ^[2]^ explored the impact of nano-topographical cues on neural cell networks. Their findings indicated that specific nano-scale surface features can significantly enhance intercellular communication among neural cells, potentially informing the development of improved neural interfaces.

Onesto et al., in their 2019 study "Cortical-like mini-columns of neuronal cells on zinc oxide nanowire surfaces" published in *Scientific Reports* ^[3]^ demonstrated that zinc oxide nanowire surfaces can guide the formation of neuronal cells into structures resembling cortical mini-columns. This suggests potential applications in creating biomimetic neural tissues for research and therapeutic purposes.

The 2020 paper "Cell Theranostics on Mesoporous Silicon Substrates" by Coluccio et al. in *Pharmaceutics* ^[33]^ investigated the use of mesoporous silicon substrates for cell theranostics. They concluded that these substrates could serve as effective platforms for both therapeutic and diagnostic applications, owing to their favorable interactions with cells.

In "Cell aggregation on nanorough surfaces," published in the *Journal of Biomechanics* in 2021, Gentile ^[34]^ examined how nanorough surfaces influence cell aggregation. The study found that such surfaces promote cell clustering, which could have implications for tissue engineering and the development of biomaterials.

Onesto et al., in their 2020 *Neural Regeneration Research* article "Small-world networks of neuroblastoma cells cultured in three-dimensional polymeric scaffolds featuring multi-scale roughness," ^[35]^ investigated the effects of multi-scale roughness in 3D polymeric scaffolds on neuroblastoma cell networks. They discovered that these scaffolds promote the formation of small-world networks, which are known for efficient information processing.

The 2016 study "Information in a Network of Neuronal Cells: Effect of Cell Density and Short-Term Depression" by Onesto et al. in *BioMed Research International* ^[36]^ analyzed how cell density and synaptic short-term depression affect information transmission in neuronal networks. The results indicated that both factors play crucial roles in modulating network efficiency and information flow.

Limongi et al., in their 2013 paper "Nanostructured Superhydrophobic Substrates Trigger the Development of 3D Neuronal Networks" in *Small* ^[37]^ demonstrated that superhydrophobic nanostructured substrates can induce the formation of three-dimensional neuronal networks. This finding offers insights into the design of biomaterials for neural tissue engineering.

In the 2013 *Scientific Reports* article "Selective modulation of cell response on engineered fractal silicon substrates," Gentile et al. explored how engineered fractal patterns on silicon substrates can modulate cell behavior ^[38]^. They found that specific fractal geometries can selectively influence cell adhesion and proliferation, providing a tool for controlling cell responses in biomedical applications.

Onesto et al., in their 2018 *Integrative Biology* paper "The effect of connectivity on information in neural networks," examined how different patterns of connectivity affect information processing in neural networks ^[39]^. Their findings suggest that optimal connectivity patterns can enhance the efficiency of neural information transmission.

In the 2019 *Journal of Physics Communications* article "Relating the small world coefficient to the entropy of 2D networks and applications in neuromorphic engineering," Onesto et al. investigated the relationship between the small-world coefficient and network entropy ^[40]^. They proposed that this relationship could inform the design of neuromorphic systems that mimic neural network architectures.

Gentile's 2021 study "Multipoint connection by long-range density interaction and short-range distance rule" in *Physica Scripta* introduced a model describing how cells establish connections based on long-range density interactions and short-range distance rules ^[41]^. This model provides insights into the principles governing cellular network formation.

In the 2022 *npj Systems Biology and Applications* paper "The small world coefficient 4.8±1 optimizes information processing in 2D neuronal networks," Aprile, Onesto, and Gentile ^[42]^ identified an optimal small-world coefficient that maximizes information processing efficiency in two-dimensional neuronal networks. This finding has potential implications for both understanding neural circuitry and designing neuromorphic systems.

Marinaro et al. ^[4]^, in their 2024 *Communications Biology* article "The role of elasticity on adhesion and clustering of neurons on soft surfaces," explored how substrate elasticity influences neuronal adhesion and clustering. They found that softer surfaces promote neuron clustering, which could inform the design of biomaterials for neural tissue engineering.

In the *Heliyon* article ^[43]^ "The maximum size of cell-aggregates is determined by the competition between the strain energy and the binding energy of cells," Gentile (2024) proposed that the size of cell aggregates is governed by a balance between mechanical strain energy and cellular binding energy. This insight enhances the understanding of tissue formation and the development of engineered tissues.

In a 2022 study published in *Frontiers in Bioengineering and Biotechnology*, Sharaf et al. ^[44]^ developed multi-scale engineered polymeric microenvironments using two-photon polymerization to better mimic the native brain tissue's softness and topography. They fabricated biomimetic 2.5D micro- and nano-pillar arrays and 3D micro-cages, which were tested with primary microglia derived from adult rhesus macaques. The results showed that microglia cultured on these structures exhibited a more ramified, homeostatic phenotype compared to those on flat substrates, suggesting that such engineered environments can provide more representative in vitro models for studying microglia in both healthy and diseased conditions.

Flamourakis et al., in their 2024 study published in *Advanced Functional Materials* ^[45]^, investigated how topographic and mechanical cues influence neuronal growth cones and network directionality. They designed nanopillar arrays using two-photon polymerization to mimic the soft, fibrous extracellular matrix of the brain. By adjusting the width and height of these pillars, they tuned their effective shear modulus, creating an environment that tricked neurons into "thinking" they were in a soft, brain-like setting. Neurons cultured on these nanopillars exhibited organized growth patterns and formed networks at specific angles, providing insights into how neurons form networks and how this process may change in neurological disorders.

These studies collectively highlight the importance of engineered microenvironments in influencing cell behavior, with applications ranging from improved in vitro models for studying brain cells to the design of medical device surfaces.

**Supporting Information 6.** *Practical implementation of AI in nano-topography enhanced topological cell analysis*.

Artificial intelligence (AI) offers powerful capabilities for identifying and linking key two-dimensional (2D) and three-dimensional (3D) topological and morphological features in vitro with cell radio-resistance. By leveraging AI, intricate patterns and correlations can be extracted from vast image datasets, facilitating deeper insights into cellular behavior and responses to radiation therapy (RT).

To analyze the relationship between topological properties of cell networks and RT characteristics, two primary approaches can be employed: machine learning (ML) and deep learning (DL). These methods are complementary, as ML requires prior identification of key network metrics derived from conventional network science algorithms, whereas DL directly processes fluorescence images of cells to extract relevant features.

*Machine Learning for Topological Cell-Network Analysis*. ML techniques integrate topological features derived from cell-graph analysis—such as mean clustering coefficient, characteristic path length, and small-world coefficient—with morphological characteristics of cell clusters, including area, perimeter, and eccentricity. Exploratory data analysis is a crucial step in understanding datasets, identifying patterns, detecting anomalies, and formulating hypotheses for further investigation. Both unsupervised and supervised ML techniques can be leveraged to extract the most relevant features contributing to specific predictive tasks, such as determining the grade of radio-resistance (RR), the amount of delivered radiation dose, and the classification of different cell lines. A range of ML models can be explored and evaluated for regression and classification tasks. To enhance model performance, feature engineering techniques—including normalization, selection, and aggregation—can be employed, optimizing predictive accuracy and robustness.

*Deep Learning for Automated Cellular Analysis*. DL approaches provide an alternative pathway for analyzing cellular networks by directly processing high-resolution fluorescence images. Convolutional Neural Networks (CNNs) can be employed to automate feature extraction and identify the most relevant image regions for predicting RR classifications. Unlike ML, which requires predefined feature extraction, DL models learn hierarchical representations directly from raw image data, offering enhanced adaptability and precision. To improve interpretability, Gradient-weighted Class Activation Maps (Grad-CAM) can be used to visualize and highlight the critical areas of cellular images that drive model predictions. This explainability facilitates the integration of DL-derived insights with ML-generated findings and domain knowledge, ensuring a comprehensive and biologically meaningful interpretation of results.

*Integrative AI-Driven Approach*. The synergy between ML and DL techniques in topological cell-network analysis holds significant potential for advancing the understanding of cellular responses to radiation. By combining conventional feature extraction methods with state-of-the-art deep learning frameworks, researchers can develop robust predictive models that enhance precision medicine strategies.

Detailed implementation strategies for ML and DL techniques in extracting key cell network metrics have been deliberately omitted, as this work is intended as a perspective article proposing strategic directions for enhancing radiation therapy assessment. Rather than providing technical methodologies, the focus is on conceptual approaches and their potential impact. The precise execution and refinement of these strategies are left to domain experts and researchers actively engaged in advancing these computational frameworks.

**References**

[1] G. Marinaro, R. La Rocca, A. Toma, M. Barberio, L. Cancedda, E. Di Fabrizio, P. Decuzzi, F. Gentile, *Integrative Biology.* **2015**, 7, 184.

[2] V. Onesto, L. Cancedda, M. L. Coluccio, M. Nanni, M. Pesce, N. Malara, M. Cesarelli, E. Di Fabrizio, F. Amato, F. Gentile, *Scientific reports.* **2017**, 7, 9841.

[3] V. Onesto, M. Villani, R. Narducci, N. Malara, A. Imbrogno, M. Allione, N. Costa, N. Coppedè, A. Zappettini, C. V. Cannistraci, *Scientific reports.* **2019**, 9, 4021.

[4] G. Marinaro, L. Bruno, N. Pirillo, M. L. Coluccio, M. Nanni, N. Malara, E. Battista, G. Bruno, F. De Angelis, L. Cancedda, *Communications Biology.* **2024**, 7, 617.

[5] N. O. Monteiro, J. F. Fangueiro, R. L. Reis, N. M. Neves, *Bioactive Materials.* **2023**, 28, 337.

[6] T. Bhuvanesh, Y. Nie, R. Machatschek, N. Ma, A. Lendlein, *Advanced Functional Materials.* **2023**, 33, 2304268.

[7] C. Jensen, Y. Teng, *Frontiers in molecular biosciences.* **2020**, 7, 33.

[8] L. Tirinato, V. Onesto, D. Garcia-Calderon, F. Pagliari, M. Spadea, J. Seco, F. Gentile, *Ann. Biomed. Eng.* **2023**, 51, 1859.

[9] L. Tirinato, V. Onesto, D. Garcia-Calderon, F. Pagliari, M. Spadea, J. Seco, F. Gentile, *Scientific reports.* **2022**, 12, 12980.

[10] Q. Akolawala, A. Accardo, *ACS Applied Materials & Interfaces.* **2025**.

[11] N. Brix, D. Samaga, R. Hennel, K. Gehr, H. Zitzelsberger, K. Lauber, *Radiation Oncology.* **2020**, 15, 1.

[12] R. A. Koch, M. Boucsein, S. Brons, M. Alber, E. Bahn, *Clinical and Translational Radiation Oncology.* **2023**, 42, 100662.

[13] T. Oike, S. Komatsu, Y. Komatsu, A. Nachankar, N. D. M. Darwis, A. Shibata, T. Ohno, *J. Radiat. Res.* **2020**, 61, 828.

[14] T. Matsui, E. Nuryadi, S. Komatsu, Y. Hirota, A. Shibata, T. Oike, T. Nakano, *International journal of molecular sciences.* **2019**, 20, 4148.

[15] N. A. Franken, H. M. Rodermond, J. Stap, J. Haveman, C. Van Bree, *Nature protocols.* **2006**, 1, 2315.

[16] L. Bodgi, H. F. Bahmad, T. Araji, J. Al Choboq, J. Bou-Gharios, K. Cheaito, Y. H. Zeidan, T. Eid, F. Geara, W. Abou-Kheir, *Frontiers in oncology.* **2019**, 9, 153.

[17] J. Luo, M. Walker, Y. Xiao, H. Donnelly, M. J. Dalby, M. Salmeron-Sanchez, *Bioactive materials.* **2022**, 15, 145.

[18] B. Geiger, A. Bershadsky, R. Pankov, K. M. Yamada, *Nature reviews Molecular cell biology.* **2001**, 2, 793.

[19] P. Kanchanawong, G. Shtengel, A. M. Pasapera, E. B. Ramko, M. W. Davidson, H. F. Hess, C. M. Waterman, *Nature.* **2010**, 468, 580.

[20] B. Geiger, J. P. Spatz, A. D. Bershadsky, *Nature reviews Molecular cell biology.* **2009**, 10, 21.

[21] R. L. Bighetti-Trevisan, L. O. Almeida, J. I. R. Ramos, G. P. Freitas, F. S. Oliveira, J. A. R. Gordon, C. E. Tye, G. S. Stein, J. B. Lian, J. L. Stein, *Biomaterials Advances.* **2025**, 168, 214128.

[22] L. Lv, Y. Liu, P. Zhang, X. Bai, X. Ma, Y. Wang, H. Li, L. Wang, Y. Zhou, *International Journal of Nanomedicine.* **2018**, 5605.

[23] M. Pogribna, G. Hammons, *Journal of Nanobiotechnology.* **2021**, 19, 1.

[24] M. Falk, E. Lukášová, S. Kozubek, *Biochimica et Biophysica Acta (BBA)-Molecular Cell Research.* **2008**, 1783, 2398.

[25] L. F. Flores, B. R. Tader, E. J. Tolosa, A. N. Sigafoos, D. L. Marks, M. E. Fernandez-Zapico, *Cells.* **2021**, 10, 2624.

[26] A. Carlos-Reyes, M. A. Muñiz-Lino, S. Romero-Garcia, C. López-Camarillo, O. N. Hernández-de la Cruz, *Frontiers in Oncology.* **2021**, 11, 718636.

[27] S. Bansal, S. Khandelwal, L. A. Meyers, *BMC Bioinformatics.* **2009**, 10, 1.

[28] K. Erciyes, *Computation.* **2023**, 11, 188.

[29] D. Hao, C. Ren, C. Li, *BMC systems biology.* **2012**, 6, 1.

[30] Y. He, Z. J. Chen, A. C. Evans, *Cerebral cortex.* **2007**, 17, 2407.

[31] S. C. Fischer, G. W. Bassel, P. Kollmannsberger, *Journal of the Royal Society Interface.* **2023**, 20, 20230115.

[32] F. Gentile, L. Tirinato, E. Battista, F. Causa, C. Liberale, E. M. di Fabrizio, P. Decuzzi, *Biomaterials.* **2010**, 31, 7205.

[33] M. L. Coluccio, V. Onesto, G. Marinaro, M. Dell’Apa, S. De Vitis, A. Imbrogno, L. Tirinato, G. Perozziello, E. Di Fabrizio, P. Candeloro, *Pharmaceutics.* **2020**, 12, 481.

[34] F. Gentile, *J. Biomech.* **2021**, 115, 110134.

[35] V. Onesto, A. Accardo, C. Vieu, F. Gentile, *Neural regeneration research.* **2020**, 15, 759.

[36] V. Onesto, C. Cosentino, E. Di Fabrizio, M. Cesarelli, F. Amato, F. Gentile, *BioMed research international.* **2016**, 2016, 2769698.

[37] T. Limongi, F. Cesca, F. Gentile, R. Marotta, R. Ruffilli, A. Barberis, M. Dal Maschio, E. M. Petrini, S. Santoriello, F. Benfenati, *Small.* **2013**, 9, 402.

[38] F. Gentile, R. Medda, L. Cheng, E. Battista, P. E. Scopelliti, P. Milani, E. A. Cavalcanti-Adam, P. Decuzzi, *Scientific Reports.* **2013**, 3, 1461.

[39] V. Onesto, R. Narducci, F. Amato, L. Cancedda, F. Gentile, *Integrative Biology.* **2018**, 10, 121.

[40] V. Onesto, M. Romano, F. Gentile, F. Amato, *Journal of Physics Communications.* **2019**, 3, 095011.

[41] F. Gentile, *Phys. Scripta.* **2021**, 96, 045004.

[42] F. Aprile, V. Onesto, F. Gentile, *NPJ Systems Biology and Applications.* **2022**, 8, 4.

[43] F. Gentile, *Heliyon.* **2024**, 10.

[44] A. Sharaf, B. Roos, R. Timmerman, G. Kremers, J. J. Bajramovic, A. Accardo, *Frontiers in Bioengineering and Biotechnology.* **2022**, 10, 926642.

[45] G. Flamourakis, Q. Dong, D. Kromm, S. Teurlings, J. van Haren, T. Allertz, H. Smeenk, F. M. de Vrij, R. P. Tas, C. S. Smith, *Advanced Functional Materials.* **2024**, 2409451.
